# Supplementary material for: Fostering active living and healthy eating through understanding physical activity and dietary behaviours of Arabic-speaking adults: a cross-sectional study from the Middle East
Source: BMJ Open. 2018 Apr 20;8(4):e019980. doi: 10.1136/bmjopen-2017-019980 (PMC5914903; doi:10.1136/bmjopen-2017-019980)
Supplement: Author's manuscript [file bmjopen-2017-019980.draft_revisions.pdf]

BMJ Open

BMJ Open is committed to open peer review. As part of this commitment we make the peer review history of every article we publish publicly available.

When an article is published we post the peer reviewers' comments and the authors' responses online. We also post the versions of the paper that were used during peer review. These are the versions that the peer review comments apply to.

The versions of the paper that follow are the versions that were submitted during the peer review process. They are not the versions of record or the final published versions. They should not be cited or distributed as the published version of this manuscript.

BMJ Open is an open access journal and the full, final, typeset and author-corrected version of record of the manuscript is available on our site with no access controls, subscription charges or pay-per-view fees (http://bmjopen.bmj.com).

If you have any questions on BMJ Open's open peer review process please email info.bmjopen@bmj.com

BMJ Open

Fostering active living and healthy eating through understanding physical activity and dietary behaviours of Arabic-speaking adults – a cross-sectional study from the Middle East

Journal:	*BMJ Open*
Manuscript ID	bmjopen-2017-019980
Article Type:	Research
Date Submitted by the Author:	06-Oct-2017
Complete List of Authors:	Donnelly, Tam Truong; University of Calgary, Nursing/Medicine<br>Fung, Tak; University of Calgary,<br>Al-Thani, Al-Anoud ; Ministry of Public Health
**Primary Subject Heading**:	Global health
Secondary Subject Heading:	Public health, Health services research, Sports and exercise medicine, Nutrition and metabolism
Keywords:	Physical activity, Dietary behaviour, Healthy lifestyle, Arabic-speaking adults, Qatar, Middle East

SCHOLARONE™
Manuscripts

For peer review only

Fostering active living and healthy eating through understanding physical activity and dietary behaviours of Arabic-speaking adults – a cross-sectional study from the Middle East

Authors:

Tam Truong Donnelly, Tak Fung, Al-Anoud bint Mohammad Al-Thani

Institutions:

Tam Truong Donnelly, PhD
University of Calgary
Faculty of Nursing and Medicine
2500 University Dr. NW
Calgary, Alberta, Canada
T2N 1N4

Tak Fung, PhD
University of Calgary
Math/Information Technology
2500 University Dr. NW
Calgary, Alberta, Canada
T2N 1N4

Al-Anoud bint Mohammad Al-Thani, MD
Manager, Health Promotion & NCD
Public Health Department
P.O. Box 42
Doha, Qatar

Corresponding author:

Tam Truong Donnelly, PhD.
University of Calgary
Faculty of Nursing and Medicine
PF 2234, 2500 University Drive NW Calgary. Alberta. Canada T2N 1N4
Tel: 1-403-220-6867
Fax: 1-403-284-4803
Email: tdonnell@ucalgary.ca

For peer review only

Abstract

Objectives

Physical inactivity and unhealthy diets increase the risk for diabetes, cardiovascular diseases, and cancer. Many people in Qatar are sedentary and consume diets high in fats, salt and sugar. The purpose of this study was to determine physical activity levels and food habits among Arab adults living in Qatar.

Setting

A cross-sectional community-based survey was conducted with 1606 Arab adults ≥18 years of age from March 2013 to June 2015. Using non-probability sampling technique, participants were recruited from 3 universities and 7 primary healthcare centers in Qatar. Participants were interviewed using a structured survey questionnaire. Surveys included demographic, clinical characteristics, questions assess the participant's daily dietary practice. Physical activity level was assessed by the Arabic version of the International Physical Activity Questionnaire. Statistical analysis was performed using SPSS version 22.0.

Results

Of 1606 participants, 50.1% were male and 49.9% were female. The participants' mean (SD) body mass index was 28.03(5.85) Kg/m^2. Two third of participants were either overweight (36.4%) or obese (33.6%). Within the last 7 days, 64% and 39.9% did not engage in vigorous or moderate physical activity, respectively. Within the last 7 days, the mean (SD) time for vigorous physical activity was 31.12 (59.28) minutes, 46.87 (63.01) minutes for moderate physical activity, and 42.01 (47.04) minutes for walking. One third of participants ate fresh fruits and vegetables once or more times daily, and consumed fish, beef, or chicken 2-4 times a week. One fourth of participants ate pasta, cakes, or pastries 2-4 times per week and 40.6 % ate white bread daily.

Conclusions

Participants were not sufficiently active and exhibited poor dietary behavior. There is a need for a nationwide health promotion program to promote a healthier lifestyle. The information from this study can inform public health policies, programs and services in the Middle East.

Keywords

Physical activity, Dietary behaviour, Healthy lifestyle, Arabic-speaking adults, Qatar, Middle East

For peer review only

Article summary:

Strengths and limitations:

- The information reported in this paper will increase healthcare providers and researchers' awareness of the physical activity level and dietary practice among Arabic-speaking adults in Qatar.

- The study findings will be helpful to develop health and wellness programs and services that promote healthy lifestyle among Arabic-speaking adults living in Qatar and Middle East region.

- Non-probability convenience sampling limits the ability to generalize results from this study.

- This study only report data pertaining to Arabic-speaking adult participants. Younger participants (age<18) may give another view of perception in regard to their physical activity and dietary behavior.

For peer review only

Introduction

Physical inactivity is a major health and economic problem worldwide, and is an important modifiable risk factor for non-communicable diseases such as type 2 diabetes, cardiovascular disease, and some cancers [1, 2]. Physical inactivity costs health-care systems $53.8 billion worldwide in 2013 [2]. In Qatar, the annual direct and indirect cost attributable to physical inactivity was an estimated $60.7 million in 2013 [1]. A recent study categorized 9 modifiable risk factors that accounted for more than 90% of the risk for acute myocardial infarction: regular physical activity, history of hypertension, diabetes, smoking, abdominal obesity, daily consumption of fruits and vegetables, psychosocial factors, regular alcohol consumption, and a raised apolipoprotein B/ApoA1 ratio [3]. These risk factors were associated with heart attack irrespective of gender or age. According to the World Health Organization's (WHO) [4] guidelines for physical activity, healthy adults 18-64 years of age should accumulate at least 150 minutes of moderate intensity aerobic activity per week, or 75 minutes of vigorous intensity, or an equivalent combination of moderate and vigorous-intensity aerobic activity in bouts of 10 minutes or more. Results of a study in the Gulf Cooperation Council countries reported that only 39.0% to 42.1% of men and 26.3% to 28.4% of women were physically active for at least 150 minutes per week [5].

A better understanding of factors underlying health perceptions and behaviors is needed to capitalize on cardiovascular and other chronic diseases preventive efforts. Ramirez et al. [6] observed that 1 in 5 patients with the highest cardiac risk did not perceive the need to improve their physical health. Globally, unhealthy diet and lack of physical activity are leading health risks of most chronic diseases such as heart disease, and diabetes [7]. A recent systematic review found significant inverse associations between fruit and vegetable intake with systolic blood pressure, abdominal obesity, triglycerides, high-density lipoprotein cholesterol, and metabolic syndrome [8]. In the Arabian Gulf countries, the most eminent risk

For peer review only

factors of non-communicable diseases involve inadequate intake of fruit and vegetables, being overweight or obese, physical inactivity, high blood pressure, high blood cholesterol, and tobacco use [9].

Lifestyle changes such as increased physical activity, decreased television watching, and healthy dietary changes are independently associated with weight reduction [10]. Urbanization alongside the modernization and the adoption of Western culture in Arab countries have shaped their meal contents, particularly intake of food (fast food, carbonated beverages) with high in sugar, fats, and carbohydrates [11-13]. The impact of these concerns on the adoption of healthy lifestyles needs to be further explored for its implications on public education and media campaigns [14, 15]. The fundamental cause of obesity and overweight is an energy imbalance between calories consumed and calories burn up [16]. According to WHO [16], more than 1.9 billion adults were overweight in 2014; of those, over 600 million adults were obese worldwide. The highest prevalence of insufficient physical activity (31%) located in Eastern Mediterranean Region. In Qatar, the prevalence of insufficient physical activity in adults has been reported as 41.6% [17]. However, there is no specific information on the level of physical activity and dietary habits among adults in Qatar. The main purpose of this study was to determine the current physical activity levels and food habits among Arabic speaking adults living in the State of Qatar. Situated on the Gulf coast of the Arabian Peninsula (Figure 1), with a population of nearly 2.6 million [18]. According to the Institute of Chartered Accountants in England and Wales (ICAEW), the State of Qatar has the fastest growing economic in the Gulf region (total GDP growth at a forecast 3.5% in 2016 and 3.7% in 2017.) and the highest gross domestic product per capita due to its abundant oil and natural gas revenues [19].

Figure 1. Gulf coast of the Arabian Peninsula

For peer review only

Methods

Participants

A cross-sectional, community-based survey with Arabic speaking adults living in Qatar was conducted from March 2013 to June 2015. Participants were eligible for inclusion in the study if they were (1) 18 years or older, (2) self-identified as an Arabic-speaker, (3) born and/or raised in Qatar, or lived in Qatar for at least 5 years, and (4) willing to commit a minimum of 60 minutes for the questionnaire. Using non-probability convenient sampling, we approached and recruited participants at different times of the days, weeks, or months of the year at designated data collection sites – 3 universities and seven health care centers in the capital Doha, South and North of Qatar, to ensure diversity of participants that can closely represent the general Arab population in Qatar.

According to the Qatar census (2010) data, there were 1,008,937 women and men (i.e., 843,441 males and 165,496 females) between the ages of 15 to 75+ living in Doha, Al Wakra (South of Qatar), Al Khor, and Al Thakhira (North of Qatar). Based on Cochran's formula for sample size determination, a sample size of 781 women and 784 men to obtain a representative sample for 843,441 men and 165,496 females aged 15 to 75+ living in the three regions mentioned above, and using a margin of errors of 3.5% (95% confidence interval) [20]. Please note that we did not have access to census data that included only people 18 years of age and older.

Ethical approvals were obtained from Hamad Medical Corporation/Weill Cornell Institutional Review Board (IRB NO: 13-00051), the University of Calgary's Conjoint Health Research Ethics Board (REB13-0347), Qatar Primary Health Care Research Committee (Reference NO: PHCC/RC/14/02/004), Qatar University (QU-IRB 244-E/13), College of North Atlantic in Qatar (CNAQ Approval NO: 2015-3), and Qatar Supreme Council of Health (SCH-A-UCQ-050). Because little research has been conducted in Qatar, participants

For peer review only

may not be familiar with the research process. The research team carefully explained the project, to enhance accessibility, all project information was translated into Arabic and was available in both Arabic and English. Prior to the start of the study, an introductory letter written in both Arabic and English was sent to the university and community health clinic sites. Formal and informal presentations were made to all staff at the universities and community health clinics at the start of the project. Written informed consent was waived but verbal consent to participate in the study was obtained from each participant. Participants were assured that all information would be confidential. No incentive was given to participants of the study.

Data Collection

Data collection was carried out using a structured survey questionnaire, especially designed for this study. Survey instrument was developed based on literature reviewed. The survey questionnaire has four sections. The first section consists of questions asking for demographic information from participants. Section two contains questions regarding levels of physical activity using the International Physical Activity questionnaire (IPAQ) [21], section three assess participant's current and previous history of chronic diseases and finally the fourth section consists of questions assess the contents of participant's daily food intake.

All study materials were translated into Arabic and back translated into English to ensure lexical equivalence. To ensure the questionnaire items were relevant to Arabic population in Qatar, we conducted a pilot study using six focus groups stratified by age (18-30 years, 31-50 years, 51+ years) and gender (males, females). We conducted separate focus groups for men and for women to be culturally appropriate and stratified by age to group people who were likely have similar life experiences. Following the focus groups, the questionnaire was further pilot tested with 12 men and 12 women recruited from both urban

For peer review only

and semi-urban settings, and who represented different age groups across the lifespan. We further refined the data collection protocol and the questionnaire based on this pilot test. Experienced and especially trained bi-lingual (Arabic and English) research assistants conducted structured interviews in Arabic.

Body Mass Index. Obesity status was determined by the Body Mass Index (BMI). BMI represents the standard method used by both the WHO and the governmental health sectors of the majority of nations to determine whether a person is obese or overweight. BMI is determined by dividing the weight measured in kilograms of an individual by his/her height measured in meters squared. Obesity and overweight was classified on the basis of BMI as Underweight ≤ 18.5 kg/m^2, Normal Weight 18.5 – 24.9 kg/m^2, Overweight 25 – 29.9 kg/m^2, Obese Class-I 30 – 34.9 kg/m^2, Obese Class-II 35 – 39.9 kg/m^2, Obese Class-III ≥ 40 kg/m^2 [22].

Physical Activity. IPAQ asks participants to recall their physical activities in the last 7 days. The results allow researchers to classify participants into one of three levels of physical activity (i.e., low, moderate, or high). IPAQ has evidence for reliability and validity for monitoring levels of physical activity among adults 18-65 years and in diverse settings [19-21]. For this study, we used the short telephone version, which was translated into Arabic. The IPAQ website provides protocols related to data cleaning, processing, scoring, and translation [22].

Dietary Behavior. Dietary behavior was reviewed based on data collected using questionnaire. The questionnaire provides details about daily consumption for various food groups including fruits, vegetables, proteins (fish, beef, lamb or chicken), carbohydrates (bread, cereals, or pasta), milk products and other food items (soft drinks, juices or nuts) consumption, including number of servings per day. Health status was reviewed based on data collected using questionnaire. The questionnaire also included questions asking for

For peer review only

history of chronic diseases such as hypertension, diabetes, dyslipidemia, stroke, obesity, history of fractures or any allergy.

Data Analysis

Data analyses were conducted by a senior biostatistician using SPSS (IBM SPSS Statistics 22.0). Descriptive statistics analyses (frequencies, means, and standard deviations (SD) for interval variables, frequency, and proportion for categorical variables were performed for the study variables. Data was expressed in frequencies for questionnaire responses calculated for all variables in numbers and percentages. Mann-Whitney test was used to compare differences between two groups with ordinal data, Chi -Square test was used to compare difference between two groups with categorical data and independent sample t-test was used to compare group differences with interval data.

Results

Participants Characteristics

Overall, 1606 participants who met the study's inclusion criteria participated, of which 804 (50.1%) were males and 802 (49.9%) were females. The majority of study participants were under the age of 60 years. In the younger age group of 40-49 years there were approximately 20.8% participants. Almost all participants were Muslim (99.1%). More than half of the study participants were married (59.7%), and 40.3% were without a spouse. A total of 30.3% of the study's participants were Qatari nationals. Close to half of participants (43.8%) came from North African countries (Egypt, Libya, Tunisia, Algeria, Morocco, Mauritania). Citizens of the Levant countries (Syria, Lebanon, Palestine, Jordan) constituted 19.9%. The rest of participants were from the Gulf Cooperation Council (GCC) and other countries. Overall, more than one-third of participants were university (37.8%), and high school (42%) educated. Nearly one third of the participant's (32.7%) household income

For peer review only

ranged US $2746-$5491 (QAR 10,000-19,999) per month. Most participants were nonsmokers (79.1%). The majority of participants were residents of capital city, Doha (77.9%), Al-Wakra (9.4%), and Al-Khor (12.6%, Table 1).

Table 1. Selected Demographic Characteristics of Participants (n = 1606)

Characteristics	Total n (%)	Male n (%)	Female n (%)
Age (years)[a]			
18-29	517 (32.3)	261 (32.6)	256 (32)
30-39	557 (34.8)	258 (32.3)	299 (37.4)
40-49	333 (20.8)	145 (18.1)	188 (23.5)
50-59	136 (8.5)	90 (11.3)	46 (5.8)
60-69	49 (3.1)	39 (4.9)	10 (1.3)
70+	8 (0.5)	7 (0.9)	1 (0.1)
Marital Status			
Single/never married	595 (37)	307 (38.2)	288 (35.9)
Married	958 (59.7)	487 (60.6)	471 (58.7)
Separated/Divorced/Widowed	53 (3.3)	10 (1.2)	43 (5.4)
Nationality			
Qatari	487 (30.3)	190 (23.6)	297 (37.1)
North Africa	704 (43.8)	415 (51.7)	289 (36)
Levant	320 (19.9)	159 (19.7)	161 (20.1)
Other GCC	33 (2)	12 (1.5)	21 (2.6)
Other	61 (3.8)	28 (3.4)	33 (4.2)
Level of Education[b]			
Never went to school	18 (1.1)	8 (1)	10 (1.2)
Primary school	115 (7.2)	45 (5.6)	70 (8.7)
High school	685 (42)	330 (41.1)	355 (44.3)
Trade school	34 (2.1)	26 (3.2)	8 (1)
University	607 (37.8)	319 (39.7)	288 (36)
Other	145 (9)	75 (9.3)	70 (8.7)
Monthly Income in USD[c]			
les that $274	7 (0.6)	5 (0.8)	2 (0.3)
$275- $2745	192 (15.7)	103 (16.9)	89 (14.5)
$2746- $5491	401 (32.7)	224 (36.7)	177 (28.8)
$5492- $8237	292 (23.8)	140 (23)	152 (24.7)
$8238- $10983	133 (10.9)	57 (9.3)	76 (12.4)
$10984- $13729	73 (6)	26 (4.3)	47 (7.6)
$13730 - $16476	49 (4)	22 (3.6)	27 (4.4)
more than $16476	78 (6.4)	33 (5.4)	45 (7.3)
Smoking[d]			
Yes	334 (20.9)	293 (36.8)	41 (5.1)

No	1263 (79.1)	504 (63.2)	759 (94.9)
Living Area			
Al-Doha	1251 (77.9)	626 (77.9)	625 (77.9)
Al-Wakra	151 (9.4)	78 (9.7)	73 (9.1)
Al-Khor	202 (12.6)	98 (12.2)	104 (13)
Dokhan	2 (0.1)	2 (0.2)	0 (0)

[a] 6 participant did not answer this question, [b] 2 participant did not answer this question, [c] 381 participant did not answer this question, [d] 9 participant did not answer this question.

Participants' Body Mass Index

The mean (SD) BMI of the study participants was 28.03 (5.85) kg/m^2 and ranged from 12.8 kg/m^2 to 65.44 kg/m^2. Overall prevalence's of underweight, normal weight and overweight were 44 (2.8%), 433 (27.2%), and 580 (36.4%) respectively. Obesity class I, II and III were observed in 383 (24%), 103 (6.5%) and 50 (3.1%) respectively (Fig 2).

Figure 2. Distribution of BMI among participants

Nearly one third of participants, were obese. The majority of obese participants were classed as Obese Class I (24.2% male and 23.9% female). Thirty-four per cent of the female participants and 38.8% of male participants were overweight. It is important to note that more female than male were in the underweight and obese class III categories based on their BMI.

Level of Physical Activity

Sixty four percent of study participants did not perform any vigorous physical activity and 39.9% did not perform any moderate physical activity during last 7 days (Table 2). Within the last 7 days, participants spent a mean (SD) of 31.12 (59.28) minutes doing vigorous physical activity, 46.87 (63.01) minutes doing moderate physical activity, and 42.01 (47.04) minutes walking. Male participants spent significantly ($p < .001$) longer time doing vigorous physical activity (mean rank score 859) and walking (mean rank score 831.1) compared to females (mean rank score 690.6, mean rank score 730.5, respectively). However,

For peer review only

males (mean rank score 769.5) and females (mean rank score 789.5) spend similar time doing moderate physical activity (p = .361). Participant spent a mean (SD) of 7.39 (3.36) hours sitting per weekday. Approximately half of participants (53.2%) spent 5-10 hours sitting, 30.3% spent 1-5 hours sitting, and 16.2% spent 10-16 hours sitting per weekday. Only 0.3 % of participants reported spent zero hours sitting per weekday.

For peer review only

Table 2. Participants Report of their Physical Activity Level during 7 days (n =1606)

Physical Activities	*None n (%)*	*1 day n (%)*	*2 days n (%)*	*3 days n (%)*	*4 days n (%)*	*5 days n (%)*	*6 days n (%)*	*7 days n (%)*
Vigorous physical activities [a]	987 (64)	120 (7.8)	123 (8)	114 (7.4)	49 (3.2)	54 (3.5)	23 (1.5)	71 (4.6)
Moderate physical activities [b]	622 (39.9)	174 (11.2)	186 (11.9)	186 (11.9)	45 (2.9)	96 (6.2)	38 (2.4)	210 (13.5)
Walk for at least 10 minutes at a time [c]	272 (17.4)	211 (13.5)	226 (14.4)	209 (13.3)	68 (4.3)	234 (14.9)	79 (5)	268 (17.1)

[a] 65 participants did not answer this question, [b] 49 participants did not answer this question,
[c] 39 participants did not answer this question.

For peer review only

Most of the 1,606 participants (98.6%) believe physical activity and eating a healthy diet are beneficial, however, less than half (45.4%) reported engaging in physical activity and only 34.5% reported eating healthy foods. Table 3 shows the participants' perceived level of physical activity and healthy eating.

Table 3. Level of Physical Activity and Healthy Eating (n=1604)

Perceived Level of Physical Activity and Healthy Eating	Yes n (%)	No n (%)	Sometimes n (%)
Do you engage in PA?	729 (45.4%)	466 (29.1%)	409 (25.5%)
Do you think you eat healthy food?	553 (34.5%)	432 (26.9%)	618 (38.5%)

Dietary Behavior

Nearly one third of participants ate fresh fruits (35.8%), green vegetables (31.8%), and other vegetables such as carrots, tomatoes, cucumber (44.1%) once or more times daily (Table 4). Similarly, nearly one third of participants consumed protein products such as fish, beef, or chicken 2-4 times a week. One fourth of the study participants drank full fat milk, 20.6% drank skimmed or low-fat milk, and nearly 50% eaten milk products more than once daily. More than one quarter of the study participants ate pasta and cakes or pastries 2-4 times in a week, 40.6% had a meal with white bread daily, and 14.4% drank cola drinks more than once per day.

Table 4. Food intake for daily meal (n =1606)

Food	Never n (%)	Seldom n (%)	1/week n (%)	2-4 /week n (%)	5-6 /week n (%)	1+ daily n (%)
Fruits & Vegetables						
Fresh fruits (apples, oranges, pears)[a]	20 (1.3)	123 (7.7)	165 (10.3)	469 (29.3)	251 (15.7)	572 (35.8)
Green leafy vegetables[b] *(lettuce, cabbage, spinach)*	35 (2.2)	99 (6.2)	180 (11.2)	485 (30.3)	293 (18.3)	510 (31.8)
Other vegetables, carrots, tomatoes, cucumber)[c]	22 (1.4)	68 (4.2)	85 (5.3)	392 (24.4)	326 (20.3)	708 (44.1)
Proteins						
Fish[d]	128 (8)	292 (18.3)	609 (38.1)	462 (28.9)	63 (3.9)	45 (2.8)
Beef[e]	354 (22.3)	352 (22.1)	334 (21)	417 (26.2)	78 (4.9)	56 (3.5)
Lamb[f]	197 (12.4)	279 (17.6)	397 (25.1)	532 (33.6)	91 (5.7)	88 (5.6)
Chicken[a]	18 (1.1)	38 (2.4)	177 (11.1)	737 (46.1)	345 (21.6)	285 (17.8)
Meat products (sausages, burgers, shawarma)[g]	276 (17.3)	496 (31.1)	341 (21.4)	305 (19.1)	110 (6.9)	67 (4.2)
Eggs[g]	102 (6.4)	209 (13.1)	277 (17.4)	580 (36.4)	176 (11)	251 (15.7)
Legumes (lentils, beans, peas)[h]	102 (6.4)	284 (17.7)	391 (24.3)	545 (33.9)	136 (8.5)	128 (8)
Milk Products						
Milk, full fat[e]	430 (27)	234 (14.7)	115 (7.2)	245 (15.4)	174 (10.9)	393 (24.7)
Milk, low fat or skimmed[i]	528 (33.1)	317 (19.9)	104 (6.5)	199 (12.4)	120 (12.5)	328 (20.6)
Milk product (cheese, yogurt, milk drinks)[j]	38 (2.4)	90 (5.6)	100 (6.2)	393 (24.5)	266 (16.6)	716 (44.7)
Carbohydrates						
Bread, white[c]	169 (10.6)	175 (10.9)	81 (5.1)	257 (16.1)	237 (14.8)	682 (42.6)
Bread, whole meal/brown[k]	432 (27)	408 (25.5)	131 (8.2)	201 (12.6)	95 (5.9)	331 (20.7)
Cereals (cornflakes, oatmeal)[l]	494 (30.9)	423 (26.5)	225 (14.1)	208 (13)	75 (4.7)	172 (10.7)
Pasta (spaghetti, macaroni, noodles, grits)[j]	72 (4.5)	201 (12.5)	387 (24.1)	469 (29.3)	202 (12.6)	272 (17)
Snack foods (potato chips, popcorn, chocolates…)[a]	211 (13.2)	385 (24.1)	307 (19.2)	368 (23)	135 (8.4)	194 (12.1)
Cakes and pastries (cakes, biscuits, sweet pies)[a]	143 (8.9)	414 (25.8)	351 (21.9)	416 (26)	126 (7.9)	150 (9.4)

Others						
Soft drinks (cola drinks)[c]	531 (33.2)	335 (20.9)	171 (10.7)	223 (13.9)	111 (6.9)	230 (14.4)
Nuts (pistachio, cashew nuts)[m]	154 (9.7)	501 (31.5)	335 (21.1)	346 (21.8)	79 (5)	174 (11)
Fruit Juices[d]	102 (6.4)	217 (13.6)	263 (16.4)	508 (31.8)	204 (12.8)	305 (19.1)
Fruit drinks[a]	310 (19.4)	321 (20.1)	131 (8.2)	373 (23.3)	158 (9.8)	307 (19.1)
Coffee/tea[c]	77 (4.8)	82 (5.1)	51 (3.2)	142 (8.9)	216 (13.5)	1033 (64.5)

[a] 6 participants did not answer this question, [b] 4 participants did not answer this question, [c] 5 participants did not answer this question, [d] 7 participants did not answer this question, [e] 15 participants did not answer this question, [f] 22 participants did not answer this question, [g] 11 participants did not answer this question, [h] 20 participants did not answer this question, [I] 10 participants did not answer this question, [j] 3 participants did not answer this question, [k] 8 participants did not answer this question, [l] 9 participants did not answer this question, [m] 17 participants did not answer this question.

For peer review only

Despite the perceived low level of being physically active (45.4%) and eating healthy (34.5%), the majority of participants found that engaging in physical activity (88.7%) and eating healthy foods (74.3%) are pleasant. Furthermore, 98.6% of participants believed that being active and eating healthy are beneficial. More than half strongly agreed that engaging in physical activity (73.1%) and eating health food (66.6%) would help them maintain good health. Out of 1606, 75.9% of participants communicated that they would engage in physical activity and 75.5% said they would eat healthy foods within the next month. Many were willing to continue to be physically active for the next 6 months (81.5%) and eat healthy foods (79.2%).

Reported Health Status

Nearly half of the study participants (49.1%) reported good health, while the remaining were of excellent (26.2%), fair (19.3%), or poor (4.5%) health. Most participants did not have a medical history of heart attack, cancer, psychiatric illness, polycystic ovarian syndrome, or fractures. Overall, 10% to 13% of the participants reported high blood cholesterol level, high blood pressure, diabetes, obesity, or bowel problems (Table 5).

Table 5. Health Status of Study Participants – (n =1606)

MEDICAL HISTORY	YES N (%)	NO N (%)
HIGH BLOOD PRESSURE [A]	206 (12.9)	1389 (87.1)
BOWEL PROBLEMS [C]	195 (12.2)	1409 (87.8)
HIGH BLOOD CHOLESTEROL [B]	183 (11.5)	1411 (88.5)
DIABETES [D]	180 (11.2)	1422 (88.8)
OBESITY [E]	162 (10.1)	1443 (89.9)
ALLERGIES [F]	146 (9.1)	1457 (90.9)
STOMACH PROBLEMS [E]	130 (8.1)	1475 (91.9)
ASTHMA [E]	73 (4.5)	1532 (95.5)
FRACTURES [E]	46 (2.9)	1559 (97.1)
POLYCYSTIC OVARIAN SYNDROME [D]	45 (2.8)	1557 (97.2)
OSTEOPOROSIS [G]	44 (2.8)	1553 (97.2)

For peer review only

PSYCHIATRIC ILLNESS [E]	12 (0.7)	1593 (99.3)
HEART ATTACK [C]	10 (0.6)	1594 (99.4)
CANCER	2 (0.1)	1604 (99.9)
STROKE [C]	1 (0.1)	1603 (99.9)

[a] 11 participants did not answer this question, [b] 12 participants did not answer this question, [c] 2 participants did not answer this question, [d] 4 participants did not answer this question, [e] 1 participants did not answer this question, [f] 3 participants did not answer this question, [g] 9 participants did not answer this question,

Discussion

In this cross-sectional community-based survey study, we examined the level of physical activity, dietary habits, and health status among adult Arabic speakers living in the State of Qatar. Our findings highlight the low physical activity levels in study participants. Studies indicated that low physical activity level in the Middle East is related to weather condition [23, 24]. The environmental factor of a hot desert climate is a barrier for people to engage in exercise and participate in outdoor activities. In summer, temperature is very high in Qatar (30–50 degree Celsius), which restricts outdoor activities such as walking, cycling, and jogging. Other reported barriers include lack of interest, motivation, and information about the benefits of exercise, stress, excessive internet and computer usage, and lack of exercise facilities [15, 25].

The findings of this study indicated that there is differences in the level of vigorous and mild (i.e. casual walking) activity between male and female participants. Previous studies have reported similar results with females being less involved in regular physical activity [26, 27]. Recent work in Qatar revealed that men were more physically active than women, both for walking (74.6% of men versus 55.3% of women) and intense sports (16.5% of men versus 8.9% of women) [28]. An Omani study found that females were significantly more inactive compared to males (76.9% versus 33.3%) [29]. Direct comparisons across studies are complicated due to multiple factors such as methodological variations, ethnicity, geographical location and cultural values [30]. However, low involvement of female in

For peer review only

regular physical activities in Qatar is possibly related to cultural and social norms as women in many Islamic countries need to be accompanied by a male family member when going outdoors, a barrier towards physical activities, and a general lack of social support for exercise [31-33].

Sitting has been highlighted as a specific marker of sedentary behaviors [34]. In this study, it was alarming that participants sat over 7 hours per week day and slightly more than half (53.2%) spent 5-10 hours sitting per week day. In contrast, recently published research reported overall median time spent in sedentary activities was 179 minutes (2.98 hours) per day among Qatari nationals [24]. More sitting time and insufficient physical activity are deleteriously and are significantly associated with higher health risk factors such as abdominal obesity, dyslipidemia, hyperglycemia and hypertension among the adult population [35].

In the present study, about two third of the participants were either overweight or obese with mean BMI of 28.03 Kg/m^2. Our findings are in consistent with a previous study conducted in Qatar, where 70.1% of participants had a BMI equal to or above 25 kg/m^2 (classified as overweight and obese) [28]. The prevalence of obesity in GCC countries is among the highest in the world. According to the Global status report in 2014 [17], rates of obesity reached more than 37% in the UAE, almost 40% in Kuwait, and more than 42% in Qatar. Similarly, high body-mass index in Saudi Arabia (34.7%) and in Lebanon (31.9%) have been reported [36].

The rapid increase in wealth and subsequent development of Arab countries has led to changes in lifestyle. One of the many consequences of region development, secondary to abundant oil resources, has been a significant change in eating habits, with the GCC's former Mediterranean-style diet of traditional products (e.g., dates, vegetables, wheat) replaced by a reliance on fast food that is dominated by refined and processed meals heavy in sugar and

other carbohydrates [37]. There is strong evidence for the benefits of healthy diet in the primary prevention of major cardiovascular events, and for reducing the risk of diabetes among people with high cardiovascular risk [38, 39]. In a recent qualitative study, Donnelly and colleagues [14] examined socio-cultural factors that influenced the healthy lifestyles (i.e., diet, physical activity, non-smoking) of 50 Arabic women with heart disease living in Qatar. The participants reported that their diets tended to be high in salt, sugar, and fats. Their diets were also influenced by traditional cultural beliefs and values. For example, women were often invited to each other homes where sweets and coffee were served. The women ate the foods that were offered because refusing to eat the food would be considered socially unacceptable behavior. Furthermore, Donnelly and colleagues [15] reported that social support, cultural values, religion, hot desert climate, heart disease, changing socio-demographic and economic conditions impacted both positively and negatively on the ability of these women to pursue a healthy lifestyle.

Overall, participant's dietary habits in this study were not found to be healthy: 40.6% ate white bread daily and had pasta and cakes or pastries 2-4 times per week. Non-carbohydrate-containing foods such as fruits, legumes, vegetables, minimally processed whole grains are healthy and cardio-metabolic protective, while foods rich in refined grains for instance white bread, white rice, crackers, cereals, bakery desserts, starches, and added sugars are associated with weight gain [10]. A study conducted in Oman reported few gender differences with respect to eating habits, except in dairy and meat consumption where 62.5% and 55.5% of males consumed more than 3 servings, compared to 18.78 % and 35.2% of females, respectively [29]. Dietary behaviors are mainly influences by individual factors (taste preferences, dietary knowledge, stress, body image, former eating habits and physical activity level) and environmental factors (availability and accessibility, costs of food products) [40]. Given that more female than male in the categories of underweight and obese

For peer review only

class III in our study, facilitating Arab women's engagement in healthier lifestyle needs to be emphasized. There is a need to develop collaboration among health care professionals, academics, public health professionals, and policy makers to improve the dietary behaviors and lifestyle of the population. In the UK, the National Institute for Health and Care Excellence (NICE) recommends that primary care practitioners deliver, brief physical activity advice to lethargic adults, and follow this up at next appointments [41].

Conclusion

Study results concluded there is insufficient level of physical activity and poor dietary behavior. Insufficient level of physical activity and unhealthy dietary present a great health risk. There is a need to develop a nationwide health promotion program aims at increase knowledge about the benefit of being physically active and eating healthy. Awareness of and adherence to the new Arabic dietary guidelines should be encourage by health care policy makers and health care providers. The findings of this study provide insights and information necessary for the development of public health policies and promotion programs in Qatar and in the Middle East region.

Study Limitations

The present study nonetheless has multiple limitations that should be considered when interpreting the results. The findings of this study may not be valid for generalization to other population segments of different race/ethnicity and socio-economic status due to geographic/cultural differences. However, the findings are communicable to the population with similar ethnic and cultural backgrounds. In this study, participants were 18 years of aged and over. Younger participants (age <18 years) may possibly give another view of perception in regard to their physical activity and dietary behaviour. In addition, information on dietary

For peer review only

habits in the present study was based on the frequency of consumption of food items without much consideration of quantity or portion size. Future research is needed to investigate the association between Arabs attitude, perceived behavioural control and their physical activity level.

Acknowledgments

This study and publications was made possible by a grant from Qatar National Research Fund under its National Priority Research Program (**NPRP 6 – 049 – 3 – 009)** for which we are very grateful. Its contents are solely the responsibility of the authors and do not necessarily represent the official views of Qatar National Research Fund.

We thank our study participants who accepted to join us and share their experiences. We hope through this report we repay them for their insight and trust. We give special thanks to the staff at universities and college, and Primary Health Care Centers who helped us to recruit the research participants, to our project coordinator, Dr. Shima Sharara and to our research assistants: Dr. Nagla HagOmer, Sara Saeed, Samira Mouhamed. We also thank Muhammad A Siddiqui for his assistance with writing this report.

Authors' contributions

TTD: Contributed to the conception and design of the study and the acquisition, analysis, and interpretation of data, drafted the manuscript, and gave final approval of the manuscript version submitted for publication.

TF: Contributed to the conception and design of the study and the acquisition of data, revised the manuscript, and gave final approval of the manuscript version submitted for publication.

AA: Contributed to the conception and design of the study and the acquisition of data, reviewed the manuscript critically for content, and gave final approval of the manuscript version submitted for publication.

Funding

This study was made possible by a grant from the Qatar National Research Fund under its National Priorities Research Program (**NPRP 6 – 049 – 3 – 009**). Its contents are solely the responsibility of the authors and do not necessarily represent the official views of the Qatar National Research Fund.

Conflict of interest

The authors report no relationships that could be construed as a conflict of interest.

Ethics approval

The ethical approvals were obtained from several ethics review boards (IRBs) which include: Hamad Medical Corporation/Weill Cornell Institutional Review Board (IRB NO: 13-00051),

the University of Calgary's Conjoint Health Research Ethics Board (REB13-0347), Qatar Primary Health Care Research Committee (Reference NO: PHCC/RC/14/02/004), Qatar University (QU-IRB 244-E/13), College of North Atlantic in Qatar (CNAQ Approval NO: 2015-3), and Qatar Supreme Council of Health (SCH-A-UCQ-050).

Data sharing statement

No additional data are available.

References

1. Ding D, Lawson K, Kolbe-Alexander T, Finkelstein E, Katzmarzyk P, van Mechelen W, et al. The economic burden of physical inactivity: a global analysis of major non-communicable diseases. The Lancet. 2016;388(10051):1311-24. doi: https://doi.org/10.1016/S0140-6736(16)30383-X.

2. Lee IM, Shiroma EJ, Lobelo F, Puska P, Blair SN, Katzmarzyk PT. Effect of physical inactivity on major non-communicable diseases worldwide: an analysis of burden of disease and life expectancy. The Lancet. 2012;380(9838):219-29. doi: https://doi.org/10.1016/S0140-6736(12)61031-9.

3. Yusuf S, Hawken S, Ôunpuu S, Dans T, Avezum A, Lanas F, et al. Effect of potentially modifiable risk factors associated with myocardial infarction in 52 countries (the INTERHEART study): case-control study. The Lancet. 2004;364(9438):937-52. doi: doi.org/10.1016/S0140-6736(04)17018-9.

4. Global recommendations on physical activity for health The World Health Organization website. . [cited 2012 October]. Available from: http://www.who.int/dietphysicalactivity/factsheet_recommendations/en/2011.

5. Mabry R, Reeves M, Eakin E, Owen N. Evidence of physical activity participation among men and women in the countries of the Gulf cooperation council: a review. Obes Rev. 2010;11(06):457-64. doi: doi: 10.1111/j.1467-789X.2009.00655.x.

6. Ramirez FD, Chen Y, Di Santo P, Simard T, Motazedian P, Hibbert B. Association Between Self□Reported Potentially Modifiable Cardiac Risk Factors and Perceived Need to Improve Physical Health: A Population□Based Study. Journal of the American Heart Association. 2017;6.

For peer review only

7. Booth FW, Roberts CK, Laye MJ. Lack of exercise is a major cause of chronic diseases. . Compr Physiol. 2012;2(2):1143–211. . doi: doi: 10.1002/cphy.c110025.

8. Collese T, Nascimento-Ferreira M, de Moraes A, Rendo-Urteaga T, Bel-Serrat S, Moreno L, et al. Role of fruits and vegetables in adolescent cardiovascular health: a systematic review. Nutr Rev. 2017;ahead of print. doi: 10.1093/nutrit/nux002.

9. World Health Organization (WHO) World Health Statistics—2014. World Health Organization. Available from: http://apps.who.int/iris/bitstream/10665/112738/1/9789240692671_eng.pdf?ua=1.

10. Mozaffarian D, Hao T, Rimm E, Willett W, Hu F. Changes in Diet and Lifestyle and LongTerm Weight Gain in Women and Men. N Engl J Med. 2011;364:2392-404.

11. Badran M, Laher I. Obesity in arabic-speaking countries. J Obes. 2011;2011:686430. doi: 10.1155/2011/686430. PubMed PMID: 22175002; PubMed Central PMCID: PMCPMC3228340.

12. Mehio Sibai A, Nasreddine L, Mokdad AH, Adra N, Tabet M, Hwalla N. Nutrition transition and cardiovascular disease risk factors in Middle East and North Africa countries: reviewing the evidence. Ann Nutr Metab. 2010;57(3-4):193-203. Epub 2010/11/23. doi: 10.1159/000321527. PubMed PMID: 21088386.

13. Ng SW, Zaghloul S, Ali H, Harrison G, Yeatts K, El Sadig M, et al. Nutrition transition in the United Arab Emirates. Eur J Clin Nutr. 2011;65(12):1328-37. Epub 2011/07/21. doi: 10.1038/ejcn.2011.135. PubMed PMID: 21772317; PubMed Central PMCID: PMCPMC3304306.

14. Donnelly TT, Al Suwaidi J, Al Bulushi A, Al Enazi N, Yassin K, Rehman AM, et al. The influence of cultural and social factors on healthy lifestyle of Arabic women. Avicenna. 2011;(2011):3. doi: 10.5339/avi.2011.3.

15. Donnelly TT, Al Suwaidi J, Al Bulushi A, Al Enazi N, Yassin K, Rehman AM, et al. Qatari women living with cardiovascular diseases-challenges and opportunities to engage in healthy lifestyles. Health Care Women Int. 2012;33(12):1114-34. doi: 10.1080/07399332.2012.712172. PubMed PMID: 23153347.

For peer review only

16. World Health Organisation: Obesity and overweight 2016. Available from: http://www.who.int/mediacentre/factsheets/fs311/en/.

17. Global status report on noncommunicable diseases. Geneva: World Health Organization. 2014. Available from: http://apps.who.int/iris/bitstream/10665/148114/1/9789241564854_eng.pdf?ua=1.

18. Qatar Statistics Authority, Ministry of Development Planning and Statistics, Population and Social Statistics. 2016.

19. Institute of Chartered Accountants in England and Wales (ICAEW) Economic Insight: Middle East. Quarterly briefing Q3. 2016. Available from: http://www.icaew.com/-/media/corporate/files/technical/economy/economic-insight/middle-east/me-q3-2016-web.ashx

20. WG C. Sampling Technique. 3rd ed ed: John Wiley & Sons: New York; 1997.

21. Craig C, Marshall A, Sjöström M, Bauman A, Booth M, Ainsworth B, et al. International physical activity questionnaire: 12-country reliability and validity. Med Sci Sports Excer. 2003;35(8):1381-95.

22. IPAQ scoring protocol. . Available from: https://sites.google.com/site/theipaq/scoring-protocol.

23. Ali HI BL, Bernsen RM. Barriers and facilitators of weight management: perspectives of Arab women at risk for type 2 diabetes. Health Soc Care Community. 2010;18(2):219-28. doi: doi: 10.1111/j.1365-2524.2009.00896.x.

24. Kahan D. Arab American college students' physical activity and body composition: reconciling Middle East-West differences using the socioecological model. Res Q Exerc Sport 2011;82(1):118-28.

25. Benjamin K, Donnelly T. Barriers and facilitators influencing the physical activity of Arabic adults: A literature review. Avicenna. 2013;2013(1):8. doi: 10.5339/avi.2013.8.

26. Al-Hazzaa HM, Al-Sobayel HI, Abahussain NA, Qahwaji DM, Alahmadi MA, Musaiger AO. Association of dietary habits with levels of physical activity and screen time

For peer review only

among adolescents living in Saudi Arabia. J Hum Nutr Diet. 2014;27 Suppl 2:204-13. doi: 10.1111/jhn.12147. PubMed PMID: 23889093.

27. Fernandes RA, Christofaro DF, Casonatto JF, Kawaguti SS, Ronque ERV, Cardoso JR, et al. Cross-sectional association between healthy and unhealthy food habits and leisure physical activity in adolescents. J Pediatr (Rio J). 2011;87(3):252-6. doi: doi:10.2223/JPED.2093.

28. Al-Thani MH A-TA, Al-Chetachi WF, Khalifa SAH, Akram H, Poovelil BV, Almalki BA, Bakri AH, Arora P, Badawi A. Dietary and Nutritional Factors Influencing Obesity in Qatari Adults and the Modifying Effect of Physical Activity. J Obes Weight-Loss Medic. 2015;1(1):1-7.

29. Kilani H, Al-Hazzaa H, Waly M, Musaiger A. Lifestyle Habits: Diet, physical activity and sleep duration among Omani adolescents. Sultan Qaboos Univ Med J. 2013;13(4):510–9.

30. Hamer M, Brunner E, Bell J, Batty G, Shipley M, Akbaraly T, et al. Physical activity patterns over 10 years in relation to body mass index and waist circumference: the Whitehall II cohort study. Obesity. 2013;21(12):E755-61.

31. Amin TT, Suleman W, Ali A, Gamal A, A. AW. Pattern, prevalence, and perceived personal barriers toward physical activity among adult Saudis in Al-Hassa, KSA. J Phys Act Health. 2011;8(6):775-84.

32. Qahoush R, Stotts N, Alawneh M, Froelicher E. Physical activity in Arab women in Southern California. Eur J Cardiovasc Nurs. 2010;9(4):263-71. doi: doi: 10.1016/j.ejcnurse.2010.03.002.

33. Shuval K, Weissblueth E, Araida A, Brezis M, Faridi Z, Ali A, et al. The role of culture, environment, and religion in the promotion of physical activity among Arab Israelis. Prev Chronic Dis. 2008;5(3):A88. doi: D - NLM: PMC2483551 EDAT- 2008/06/19 09:00 MHDA- 2008/08/08 09:00 CRDT- 2008/06/19 09:00 AID - A88 [pii] PST - ppublish.

34. Owen N, Bauman A, Brown W. Too much sitting: a novel and important predictor of chronic disease risk? Br J Sports Med. 2009;43(2):81–3.

For peer review only

35. Chu AHY, Moy FM. Joint Association of Sitting Time and Physical Activity with Metabolic Risk Factors among Middle-Aged Malays in a Developing Country: A Cross-Sectional Study. Bayer A, ed. . PLoS ONE 2013;8(4):e61723. doi: doi:10.1371/journal.pone.0061723. .

36. Confronting obesity in the Middle East cultural, social and policy challenges. The Economist Intelligence Unit Limited 2016. Available from: https://www.eiuperspectives.economist.com/sites/default/files/ConfrontingobesityintheMiddleEast_0.pdf

37. Gerber M, Hoffman R. The Mediterranean diet: health, science and society. . Br J Nutr. 2015;113(2):S4-10.

38. Estruch R, Ros E, Salas-Salvad J, Covas M, Corella D, Arós F, et al. Primary prevention of cardiovascular disease with a Mediterranean diet. N Engl J Med. 2013;368(14):1279-90. doi: doi: 10.1056/NEJMoa1200303.

39. Salas-Salvadó J, Bulló M, Estruch R, Ros E, Covas M, Ibarrola-Jurado N, et al. Prevention of diabetes with Mediterranean diets: a subgroup analysis of a randomized trial. Ann Intern Med. 2014;160(1):1-10. doi: doi: 10.7326/M13-1725.

40. Deliens T, Clarys P, De BI, Deforche B. Determinants of eating behaviour in university students: a qualitative study using focus group discussions. BMC Public Health. 2014;14(1):53.

41. National Institute for Health and Care Excellence. Physical activity: brief advice for adults in primary care. NICE Public Health Guidance 44. National Institute for Health and Care Excellence. 2013. Available from: https://www.nice.org.uk/guidance/ph44.

For peer review only

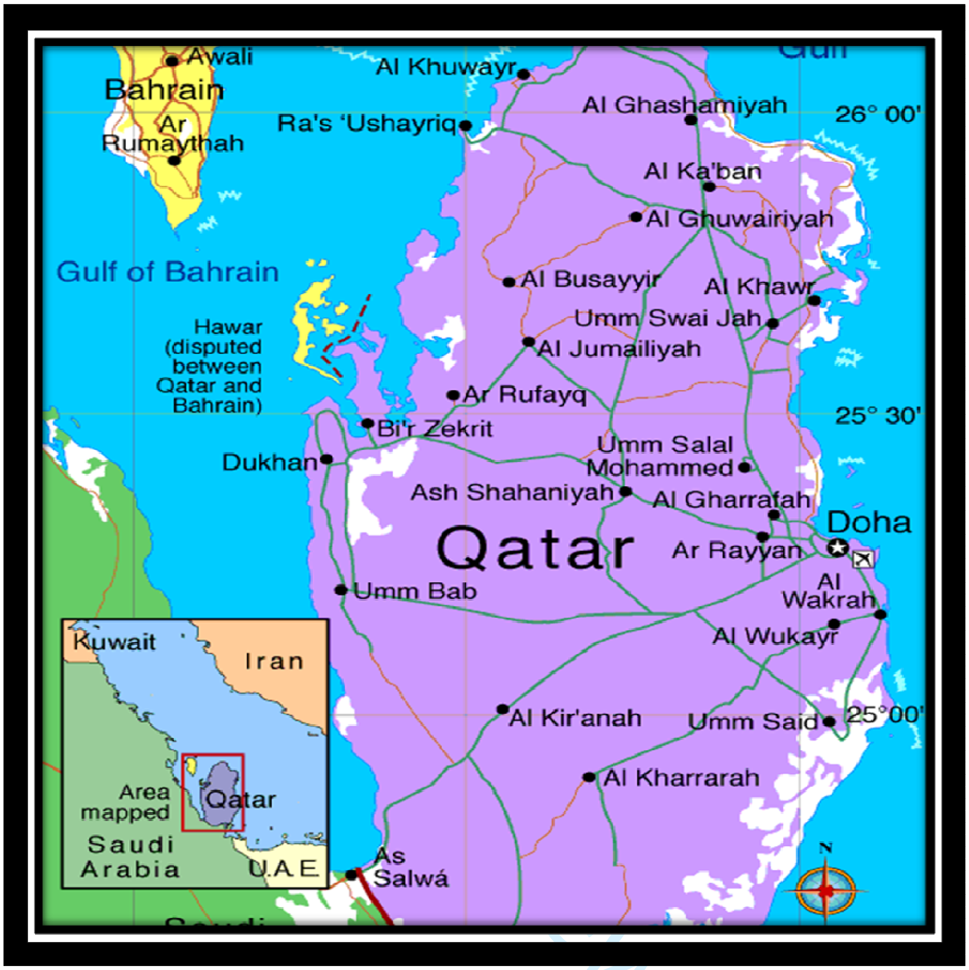

Figure 1. Gulf coast of the Arabian Peninsula

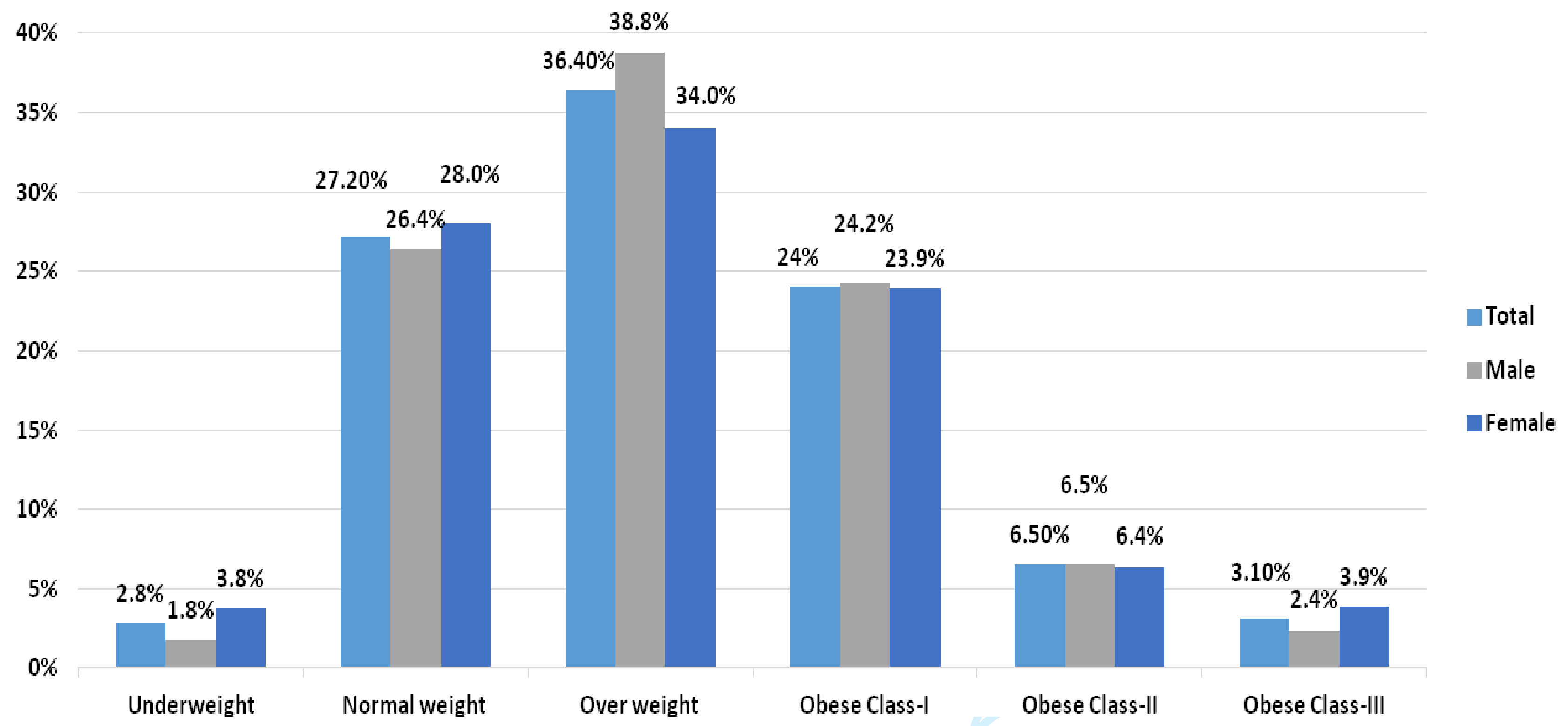

Figure 2: Distribution of BMI among participants

BMJ Open

Fostering active living and healthy eating through understanding physical activity and dietary behaviours of Arabic-speaking adults – a cross-sectional study from the Middle East

Journal:	*BMJ Open*
Manuscript ID	bmjopen-2017-019980.R1
Article Type:	Research
Date Submitted by the Author:	20-Nov-2017
Complete List of Authors:	Donnelly, Tam Truong; University of Calgary, Nursing/Medicine<br>Fung, Tak; University of Calgary,<br>Al-Thani, Al-Anoud ; Ministry of Public Health
**Primary Subject Heading**:	Global health
Secondary Subject Heading:	Public health, Health services research, Sports and exercise medicine, Nutrition and metabolism
Keywords:	Physical activity, Dietary behaviour, Healthy lifestyle, Arabic-speaking adults, Qatar, Middle East

SCHOLARONE™
Manuscripts

For peer review only

Fostering active living and healthy eating through understanding physical activity and dietary behaviours of Arabic-speaking adults – a cross-sectional study from the Middle East

Authors:

Tam Truong Donnelly, Tak Fung, Al-Anoud bint Mohammad Al-Thani

Institutions:

Tam Truong Donnelly, PhD
University of Calgary
Faculty of Nursing and Medicine
2500 University Dr. NW
Calgary, Alberta, Canada
T2N 1N4

Tak Fung, PhD
University of Calgary
Research Computing Services
Information Technologies
2500 University Dr. NW
Calgary, Alberta, Canada
T2N 1N4

Al-Anoud bint Mohammad Al-Thani, MD
Health Promotion & Non Communication Diseases
Public Health Department
P.O. Box 42
Doha, Qatar

Corresponding author:

Tam Truong Donnelly, PhD.
University of Calgary
Faculty of Nursing and Medicine
PF 2234, 2500 University Drive NW Calgary. Alberta. Canada T2N 1N4
Tel: 1-403-220-6867
Fax: 1-403-284-4803
Email: tdonnell@ucalgary.ca

For peer review only

Abstract

Objectives

Physical inactivity and unhealthy diets increase the risk for diabetes, cardiovascular diseases, and cancer. Many people in Qatar are sedentary and consume diets high in fats, salt and sugar. The purpose of this study was to determine physical activity levels and food habits among Arab adults living in Qatar.

Setting

A cross-sectional community-based survey was conducted with 1606 Arab adults ≥18 years of age from March 2013 to June 2015. Using non-probability sampling technique, participants were recruited from 3 universities and 5 primary healthcare centers in Qatar. Participants were interviewed using a structured survey questionnaire. Surveys included demographic, clinical characteristics, questions assess the participant's daily dietary practice. Physical activity level was assessed by the Arabic version of the International Physical Activity Questionnaire. Statistical analysis was performed using SPSS version 22.0.

Results

Of 1606 participants, 50.1% were male and 49.9% were female. The participants' mean (SD) body mass index was 28.03(5.85) Kg/m^2. Two thirds of participants were either overweight (36.4%) or obese (33.6%). Within the last 7 days, 64% and 39.9% did not engage in vigorous or moderate physical activity, respectively. Within the last 7 days, the mean (SD) time for vigorous physical activity was 31.12 (59.28) minutes, 46.87 (63.01) minutes for moderate physical activity, and 42.01 (47.04) minutes for walking. One third of participants ate fresh fruits and vegetables once or more times daily, and consumed fish, beef, or chicken 2-4 times a week. One fourth of participants ate pasta, cakes, or pastries 2-4 times per week and 40.6 % ate white bread daily.

Conclusions

Participants were not sufficiently active and exhibited poor dietary behavior. There is a need for a nationwide health promotion program to promote a healthier lifestyle. The information from this study can inform public health policies, programs and services in the Middle East.

Keywords

Physical activity, Dietary behaviour, Healthy lifestyle, Arabic-speaking adults, Qatar, Middle East

For peer review only

Article summary:

Strengths and limitations:

- The information reported in this paper will increase healthcare providers and researchers' awareness of the physical activity level and dietary practice among Arabic-speaking adults in Qatar.
- The study findings will be helpful to develop health and wellness programs and services that promote healthy lifestyle among Arabic-speaking adults living in Qatar and Middle East region.
- Large sample size (n=1606) and face-to-face interview with both male and female participants are major strengths of this study.
- Non-probability convenience sampling limits the ability to generalize results from this study.
- This study only report data pertaining to Arabic-speaking adult participants. Younger participants (age<18) may give another view of perception in regard to their physical activity and dietary behavior.

For peer review only

Introduction

Physical inactivity is a major health and economic problem worldwide, and is an important modifiable risk factor for non-communicable diseases such as type 2 diabetes, cardiovascular disease, and some cancers [1, 2]. Physical inactivity costs health-care systems $53.8 billion worldwide in 2013 [2]. In Qatar, the annual direct and indirect cost attributable to physical inactivity was an estimated $60.7 million in 2013 [1]. A recent study categorized 9 modifiable risk factors that accounted for more than 90% of the risk for acute myocardial infarction: sedentary lifestyle, history of hypertension, diabetes, smoking, abdominal obesity, occasional-non daily consumption of fruits and vegetables, psychosocial factors, regular alcohol consumption, and a raised apolipoprotein B/ApoA1 ratio [3]. These risk factors were associated with heart attack irrespective of gender or age. According to the World Health Organization's (WHO) [4] guidelines for physical activity, healthy adults 18-64 years of age should accumulate at least 150 minutes of moderate intensity aerobic activity per week, or 75 minutes of vigorous intensity, or an equivalent combination of moderate and vigorous-intensity aerobic activity in bouts of 10 minutes or more. Results of a study in the Gulf Cooperation Council countries reported that only 39.0% to 42.1% of men and 26.3% to 28.4% of women were physically active for at least 150 minutes per week [5].

A better understanding of factors underlying health perceptions and behaviors is needed to capitalize on cardiovascular and other chronic diseases prevention efforts. Ramirez et al. [6] observed that 1 in 5 patients with the highest cardiac risk did not perceive the need to improve their physical health. Globally, unhealthy diet and lack of physical activity are leading health risks of most chronic diseases such as heart disease, and diabetes [7]. A recent systematic review found significant inverse associations between fruit and vegetable intake with systolic blood pressure, abdominal obesity, triglycerides, high-density lipoprotein cholesterol, and metabolic syndrome [8]. In the Arabian Gulf countries, the most eminent risk

For peer review only

factors of non-communicable diseases involve inadequate intake of fruit and vegetables, being overweight or obese, physical inactivity, high blood pressure, high blood cholesterol, and tobacco use [9].

Lifestyle changes such as increased physical activity, decreased television watching, and healthy dietary changes are independently associated with weight reduction [10]. Urbanization alongside the modernization and the adoption of Western culture in Arab countries have shaped their meal contents, particularly intake of food (fast food, carbonated beverages) with high in sugar, fats, and carbohydrates [11-13]. The impact of these concerns on the adoption of healthy lifestyles needs to be further explored for its implications on public education and media campaigns [14, 15]. The fundamental cause of obesity and overweight is an energy imbalance between calories consumed and calories burn up [16]. According to WHO [16], more than 1.9 billion adults were overweight in 2014; of those, over 600 million adults were obese worldwide. The highest prevalence of insufficient physical activity (31%) located in Eastern Mediterranean Region. In Qatar, the prevalence of insufficient physical activity in adults has been reported as 41.6% [17]. However, there is no specific information on the level of physical activity and dietary habits among adults in Qatar. The main purpose of this study was to determine the current physical activity levels and food habits among Arabic speaking adults living in the State of Qatar. Situated on the Gulf coast of the Arabian Peninsula (Figure 1), with a population of nearly 2.6 million [18]. According to the Institute of Chartered Accountants in England and Wales (ICAEW), the State of Qatar has the fastest growing economic in the Gulf region (total GDP growth at a forecast 3.5% in 2016 and 3.7% in 2017.) and the highest gross domestic product per capita due to its abundant oil and natural gas revenues [19].

Figure 1. Gulf coast of the Arabian Peninsula

For peer review only

Methods

Participants

A cross-sectional, community-based survey with Arabic speaking adults living in Qatar was conducted from March 2013 to June 2015. Participants were eligible for inclusion in the study if they were (1) 18 years or older, (2) self-identified as an Arabic-speaker, (3) born and/or raised in Qatar, or lived in Qatar for at least 5 years, and (4) willing to commit a minimum of 60 minutes for the questionnaire. Using non-probability convenient sampling, we approached and recruited participants at designated data collection sites – three universities and five health care centers in the capital Doha, South and North of Qatar, to ensure diversity of participants that can closely represent the general Arab population in Qatar. Although random selection helps to reduce selection biases, this sampling technique would not be feasible for this research. It is difficult to gain access to Arabic populations because social and cultural beliefs and practices values privacy. Hence, we used a purposeful, non-probability convenient sampling technique. We realized that this will increase the risk of selection biases and will limit the generalizability of the research findings. To help offset these limitations, we recruited and randomly selected participants at different times of the days, weeks, or months of the year at the designated data collection sites (universities and community health clinics).

Trained interviewers who are fluent in both Arabic and English identified eligible participants based on the inclusion criteria. Providing the eligible individual answers “yes” to the screening questions and wishes to continue, the interviewer: (1) provided the participant with a short explanation of the study, (2) advised the participant that his/her participation is strictly voluntary, (3) advised him/her that measures that will be taken to help ensure confidentiality, and (4) answered any of the participant’s questions. If the participant agreed to participate, this was considered “consent by assent”. The interviewer then enrolled the

For peer review only

participant in the study and administered the survey. Recruitment continued until determined sample size was reached.

According to the Qatar census (2010) data, there were 1,008,937 women and men (i.e., 843,441 males and 165,496 females) between the ages of 15 to 75+ living in Doha, Al Wakra (South of Qatar), Al Khor, and Al Thakhira (North of Qatar). Based on Cochran's formula for sample size determination, a sample size of 781 women and 784 men to obtain a representative sample for 843,441 men and 165,496 females aged 15 to 75+ living in the three regions mentioned above, and using a margin of errors of 3.5% (95% confidence interval) [20]. Please note that we did not have access to census data that included only people 18 years of age and older.

Ethical approvals were obtained from Hamad Medical Corporation/Weill Cornell Institutional Review Board (IRB NO: 13-00051), the University of Calgary's Conjoint Health Research Ethics Board (REB13-0347), Qatar Primary Health Care Research Committee (Reference NO: PHCC/RC/14/02/004), Qatar University (QU-IRB 244-E/13), College of North Atlantic in Qatar (CNAQ Approval NO: 2015-3), and Qatar Supreme Council of Health (SCH-A-UCQ-050). Because little research has been conducted in Qatar, participants may not be familiar with the research process. The research team carefully explained the project, to enhance accessibility, all project information was translated into Arabic and was available in both Arabic and English. Prior to the start of the study, an introductory letter written in both Arabic and English was sent to the university and community health clinic sites. Formal and informal presentations were made to all staff at the universities and community health clinics at the start of the project. Written informed consent was waived but verbal consent to participate in the study was obtained from each participant. Participants were assured that all information would be confidential. No incentive was given to participants of the study.

For peer review only

Data Collection

Data collection was carried out using a structured survey questionnaire, especially designed for this study. Survey instrument was developed based on literature reviewed. The survey questionnaire has four sections. The first section consists of questions asking for demographic information from participants. Section two contains questions regarding levels of physical activity using the International Physical Activity questionnaire (IPAQ) [21], section three assess participant's current and previous history of chronic diseases and finally the fourth section consists of questions assess the contents of participant's daily food intake.

All study materials were translated into Arabic and back translated into English to ensure lexical equivalence. To ensure the questionnaire items were relevant to Arabic population in Qatar, we conducted a pilot study using six focus groups stratified by age (18-30 years, 31-50 years, 51+ years) and gender (males, females). We conducted separate focus groups for men and for women to be culturally appropriate and stratified by age to group people who were likely have similar life experiences. Following the focus groups, the questionnaire was further pilot tested with 12 men and 12 women recruited from both urban and semi-urban settings, and who represented different age groups across the lifespan. We further refined the data collection protocol and the questionnaire based on this pilot test. Experienced and especially trained bi-lingual (Arabic and English) research assistants conducted structured interviews in Arabic.

Body Mass Index. Obesity status was determined by the Body Mass Index (BMI). BMI represents the standard method used by both the WHO and the governmental health sectors of the majority of nations to determine whether a person is obese or overweight. BMI is determined by dividing the weight measured in kilograms of an individual by his/her height measured in meters squared. Obesity and overweight was classified on the basis of BMI as Underweight ≤ 18.5 kg/m^2, Normal Weight 18.5 – 24.9 kg/m^2, Overweight 25 – 29.9 kg/m^2,

For peer review only

Obese Class-I 30 – 34.9 kg/m^2, Obese Class-II 35 – 39.9 kg/m^2, Obese Class-III ≥ 40 kg/m^2 [22].

Physical Activity. IPAQ asks participants to recall their physical activities in the last 7 days. The results allow researchers to classify participants into one of three levels of physical activity (i.e., low, moderate, or high). IPAQ has evidence for reliability and validity for monitoring levels of physical activity among adults 18-65 years and in diverse settings [19-21]. For this study, we used the short telephone version, which was translated into Arabic. The IPAQ website provides protocols related to data cleaning, processing, scoring, and translation [22].

Dietary Behavior. Dietary behavior was reviewed based on data collected using questionnaire. The questionnaire provides details about daily or weekly frequency of consumption for various food groups including fruits, vegetables, proteins (fish, beef, lamb or chicken), carbohydrates (bread, cereals, or pasta), milk products and other food items (soft drinks, juices or nuts) consumption, including number of servings per day. For example, questions were asked: How often do you eat fresh fruits/ green leafy vegetables/ fish/ cakes and pastries, etc.? Participants can response as follow: Never; Seldom; Once a week; 2-4 times a week; 5-6 times a week; Once or more daily; Don't know.

Health status was reviewed based on data collected using questionnaire. The questionnaire also included questions asking for history of chronic diseases such as hypertension, diabetes, dyslipidemia, stroke, obesity, history of fractures or any allergy.

Data Analysis

Data analyses were conducted by a senior biostatistician using SPSS (IBM SPSS Statistics 22.0). Descriptive statistics analyses (frequencies, means, and standard deviations (SD) for interval variables, frequency, and proportion for categorical variables were performed for the study variables. Data was expressed in frequencies for questionnaire

For peer review only

responses calculated for all variables in numbers and percentages. Mann-Whitney test was used to compare differences between two groups with ordinal data, Chi -Square test was used to compare difference between two groups with categorical data and independent sample t-test was used to compare group differences with interval data.

Results

Participants Characteristics

Three thousand and eighty one (n=3081) participants were approached of which 1606 participants who met the study's inclusion criteria participated in the study (response rate 52.1%). Eight hundred and 4 (50.1%) were males and 802 (49.9%) were females. The majority of study participants were under the age of 60 years. In the younger age group of 40-49 years there were approximately 20.8% participants. Almost all participants were Muslim (99.1%). More than half of the study participants were married (59.7%), and 40.3% were without a spouse. A total of 30.3% of the study's participants were Qatari nationals. Close to half of participants (43.8%) came from North African countries (Egypt, Libya, Tunisia, Algeria, Morocco, Mauritania). Citizens of the Levant countries (Syria, Lebanon, Palestine, Jordan) constituted 19.9%. The rest of participants were from the Gulf Cooperation Council (GCC) and other countries. Overall, more than one-third of participants were university (37.8%), and high school (42%) educated. Nearly one third of the participant's (32.7%) household income ranged US $2746-$5491 (QAR 10,000-19,999) per month. Most participants were nonsmokers (79.1%). The majority of participants were residents of capital city, Doha (77.9%), Al-Wakra (9.4%), and Al-Khor (12.6%, Table 1).

For peer review only

Table 1. Selected Demographic Characteristics of Participants (n = 1606)

Characteristics	Total n (%)	Male n (%)	Female n (%)	P-values
Age (years)[a]				
18-29	517 (32.3)	261 (32.6)	256 (32)	
30-39	557 (34.8)	258 (32.3)	299 (37.4)	
40-49	333 (20.8)	145 (18.1)	188 (23.5)	
50-59	136 (8.5)	90 (11.3)	46 (5.8)	
60-69	49 (3.1)	39 (4.9)	10 (1.3)	
70+	8 (0.5)	7 (0.9)	1 (0.1)	<.001*
Marital Status				
Single/never married	595 (37)	307 (38.2)	288 (35.9)	
Married	958 (59.7)	487 (60.6)	471 (58.7)	<.001*
Separated/Divorced/Widowed	53 (3.3)	10 (1.2)	43 (5.4)	
Nationality				
Qatari	487 (30.3)	190 (23.6)	297 (37.1)	
North Africa	704 (43.8)	415 (51.7)	289 (36)	
Levant	320 (19.9)	159 (19.7)	161 (20.1)	
Other GCC	33 (2)	12 (1.5)	21 (2.6)	
Other	61 (3.8)	28 (3.4)	33 (4.2)	<.001*
Level of Education[b]				
Never went to school	18 (1.1)	8 (1)	10 (1.2)	
Primary school	115 (7.2)	45 (5.6)	70 (8.7)	
High school	685 (42)	330 (41.1)	355 (44.3)	
Trade school	34 (2.1)	26 (3.2)	8 (1)	
University	607(37.8)	319 (39.7)	288 (36)	
Other	145 (9)	75 (9.3)	70 (8.7)	0.003*
Monthly Income in USD[c]				
les that $274	7 (0.6)	5 (0.8)	2 (0.3)	
$275- $2745	192 (15.7)	103 (16.9)	89 (14.5)	
$2746- $5491	401 (32.7)	224 (36.7)	177 (28.8)	
$5492- $8237	292 (23.8)	140 (23)	152 (24.7)	
$8238- $10983	133 (10.9)	57 (9.3)	76 (12.4)	
$10984- $13729	73 (6)	26 (4.3)	47 (7.6)	
$13730 - $16476	49 (4)	22 (3.6)	27 (4.4)	
more than $16476	78 (6.4)	33 (5.4)	45 (7.3)	0.007*

Smoking [d]				
Yes	334 (20.9)	293 (36.8)	41 (5.1)	
No	1263 (79.1)	504 (63.2)	759 (94.9)	<.001*
Living Area				
Al-Doha	1251 (77.9)	626 (77.9)	625 (77.9)	
Al-Wakra	151 (9.4)	78 (9.7)	73 (9.1)	
Al-Khor	202 (12.6)	98 (12.2)	104 (13)	
Dokhan	2 (0.1)	2 (0.2)	0 (0)	0.505

[a] 6 participant did not answer this question, [b] 2 participant did not answer this question,
[c] 381 participant did not answer this question, [d] 9 participant did not answer this question.
*Significant at α=0.05 levels

Participants' Body Mass Index

The mean (SD) BMI of the study participants was 28.03 (5.85) kg/m^2 and ranged from 12.8 kg/m^2 to 65.44 kg/m^2. Overall prevalence's of underweight, normal weight and overweight were 44 (2.8%), 433 (27.2%), and 580 (36.4%) respectively. Obesity class I, II and III were observed in 383 (24%), 103 (6.5%) and 50 (3.1%) respectively (Fig 2).

Figure 2. Distribution of BMI among participants

Nearly one third of participants, were obese. The majority of obese participants were classed as Obese Class I (24.2% male and 23.9% female). Thirty-four per cent of the female participants and 38.8% of male participants were overweight. It is important to note that more female than male were in the underweight and obese class III categories based on their BMI.

Level of Physical Activity

Sixty four percent of study participants did not perform any vigorous physical activity and 39.9% did not perform any moderate physical activity during last 7 days (Table 2). Within the last 7 days, participants spent a mean (SD) of 31.12 (59.28) minutes doing vigorous physical activity, 46.87 (63.01) minutes doing moderate physical activity, and 42.01

For peer review only - http://bmjopen.bmj.com/site/about/guidelines.xhtml

(47.04) minutes walking. Male participants spent significantly ($p < .001$) longer time doing vigorous physical activity (mean rank score 859) and walking (mean rank score 831.1) compared to females (mean rank score 690.6, mean rank score 730.5, respectively). However, males (mean rank score 769.5) and females (mean rank score 789.5) spend similar time doing moderate physical activity ($p = .361$). Participant spent a mean (SD) of 7.39 (3.36) hours sitting per weekday. Approximately half of participants (53.2%) spent 5-10 hours sitting, 30.3% spent 1-5 hours sitting, and 16.2% spent 10-16 hours sitting per weekday. Only 0.3 % of participants reported spent zero hours sitting per weekday.

For peer review only

Table 2. Participants Report of their Physical Activity Level during 7 days (n =1606)

Physical Activities	***None n (%)***	***1 day n (%)***	***2 days n (%)***	***3 days n (%)***	***4 days n (%)***	***5 days n (%)***	***6 days n (%)***	***7 days n (%)***	***p-values***
Vigorous physical activities [a]	987 (64)	120 (7.8)	123 (8)	114 (7.4)	49 (3.2)	54 (3.5)	23 (1.5)	71 (4.6)	<.001*
Moderate physical activities [b]	622 (39.9)	174 (11.2)	186 (11.9)	186 (11.9)	45 (2.9)	96 (6.2)	38 (2.4)	210 (13.5)	<.001*
Walk for at least 10 minutes at a time [c]	272 (17.4)	211 (13.5)	226 (14.4)	209 (13.3)	68 (4.3)	234 (14.9)	79 (5)	268 (17.1)	<.001*

[a] 65 participants did not answer this question, [b] 49 participants did not answer this question,
[c] 39 participants did not answer this question.
All p-values are computed by one sample Chisquare test, with d.f.=7.
* Significant at α=0.05 levels.

For peer review only

Most of the 1,606 participants (98.6%) believe physical activity and eating a healthy diet are beneficial, however, less than half (45.4%) reported engaging in physical activity and only 34.5% reported eating healthy foods. Table 3 shows the participants' perceived level of physical activity and healthy eating.

Table 3. Level of Physical Activity and Healthy Eating (n=1604)

**Perceived Level of Physical Activity and Healthy Eating**	**Yes n (%)**	**No n (%)**	**Sometimes n (%)**	**P-values**
Do you engage in PA?	729 (45.4%)	466 (29.1%)	409 (25.5%)	<.001*
Do you think you eat healthy food?	553 (34.5%)	432 (26.9%)	618 (38.5%)	<.001*

All p-values are computed by one sample Chisquare test, with d.f.=2.
* Significant at α=0.05 levels.

Dietary Behavior

Nearly one third of participants ate fresh fruits (35.8%), green vegetables (31.8%), and other vegetables such as carrots, tomatoes, cucumber (44.1%) once or more times daily (Table 4). Similarly, nearly one third of participants consumed protein products such as fish, beef, or chicken 2-4 times a week. One fourth of the study participants drank full fat milk, 20.6% drank skimmed or low-fat milk, and nearly 50% eaten milk products more than once daily. More than one quarter of the study participants ate pasta and cakes or pastries 2-4 times in a week, 40.6% had a meal with white bread daily, and 14.4% drank cola drinks more than once per day.

For peer review only

Table 4. Food intake for daily meal (n =1606)

Food	Never n (%)	Seldom n (%)	1/week n (%)	2-4 /week n (%)	5-6 /week n (%)	1+ daily n (%)	P-values
Fruits & Vegetables							
Fresh fruits (apples, oranges, pears)[a]	20 (1.3)	123 (7.7)	165 (10.3)	469 (29.3)	251 (15.7)	572 (35.8)	<.001*
Green leafy vegetables[b] *(lettuce, cabbage, spinach)*	35 (2.2)	99 (6.2)	180 (11.2)	485 (30.3)	293 (18.3)	510 (31.8)	<.001
Other vegetables, carrots, tomatoes, cucumber)[c]	22 (1.4)	68 (4.2)	85 (5.3)	392 (24.4)	326 (20.3)	708 (44.1)	<.001*
Proteins							
Fish[d]	128 (8)	292 (18.3)	609 (38.1)	462 (28.9)	63 (3.9)	45 (2.8)	<.001*
Beef[e]	354 (22.3)	352 (22.1)	334 (21)	417 (26.2)	78 (4.9)	56 (3.5)	<.001*
Lamb[f]	197 (12.4)	279 (17.6)	397 (25.1)	532 (33.6)	91 (5.7)	88 (5.6)	<.001*
Chicken[a]	18 (1.1)	38 (2.4)	177 (11.1)	737 (46.1)	345 (21.6)	285 (17.8)	<.001*
Meat products (sausages, burgers, shawarma)[g]	276 (17.3)	496 (31.1)	341 (21.4)	305 (19.1)	110 (6.9)	67 (4.2)	<.001*
Eggs[g]	102 (6.4)	209 (13.1)	277 (17.4)	580 (36.4)	176 (11)	251 (15.7)	<.001*
Legumes (lentils, beans, peas)[h]	102 (6.4)	284 (17.7)	391 (24.3)	545 (33.9)	136 (8.5)	128 (8)	<.001*
Milk Products							
Milk, full fat[e]	430 (27)	234 (14.7)	115 (7.2)	245 (15.4)	174 (10.9)	393 (24.7)	<.001*
Milk, low fat or skimmed[i]	528 (33.1)	317 (19.9)	104 (6.5)	199 (12.4)	120 (12.5)	328 (20.6)	<.001*
Milk product (cheese, yogurt, milk drinks)[j]	38	90 (5.6)	100 (6.2)	393 (24.5)	266 (16.6)	716 (44.7)	<.001*

For peer review only - http://bmjopen.bmj.com/site/about/guidelines.xhtml

	(2.4)						
Carbohydrates							
Bread, white[c]	169 (10.6)	175 (10.9)	81 (5.1)	257 (16.1)	237 (14.8)	682 (42.6)	<.001*
Bread, whole meal/brown[k]	432 (27)	408 (25.5)	131 (8.2)	201 (12.6)	95 (5.9)	331 (20.7)	<.001*
Cereals (cornflakes, oatmeal)[l]	494 (30.9)	423 (26.5)	225 (14.1)	208 (13)	75 (4.7)	172 (10.7)	<.001*
Pasta (spaghetti, macaroni, noodles, grits)[j]	72 (4.5)	201 (12.5)	387 (24.1)	469 (29.3)	202 (12.6)	272 (17)	<.001*
Snack foods (potato chips, popcorn, chocolates…)[a]	211 (13.2)	385 (24.1)	307 (19.2)	368 (23)	135 (8.4)	194 (12.1)	<.001*
Cakes and pastries (cakes, biscuits, sweet pies)[a]	143 (8.9)	414 (25.8)	351 (21.9)	416 (26)	126 (7.9)	150 (9.4)	<.001*
Others							
Soft drinks (cola drinks)[c]	531 (33.2)	335 (20.9)	171 (10.7)	223 (13.9)	111 (6.9)	230 (14.4)	<.001*
Nuts (pistachio, cashew nuts)[m]	154 (9.7)	501 (31.5)	335 (21.1)	346 (21.8)	79 (5)	174 (11)	<.001*
Fruit Juices[d]	102 (6.4)	217 (13.6)	263 (16.4)	508 (31.8)	204 (12.8)	305 (19.1)	<.001*
Fruit drinks[a]	310 (19.4)	321 (20.1)	131 (8.2)	373 (23.3)	158 (9.8)	307 (19.1)	<.001*
Coffee/tea[c]	77 (4.8)	82 (5.1)	51 (3.2)	142 (8.9)	216 (13.5)	1033 (64.5)	<.001*

[a] 6 participants did not answer this question, [b] 4 participants did not answer this question, [c] 5 participants did not answer this question, [d] 7 participants did not answer this question, [e] 15 participants did not answer this question, [f] 22 participants did not answer this question, [g] 11 participants did not answer this question, [h] 20 participants did not answer this question, [l] 10 participants did not answer this question, [j] 3 participants did not answer this question, [k] 8 participants did not answer this question, [l] 9 participants did not answer this question, [m] 17 participants did not answer this question.

All p-values are computed by one sample Chisquare test, with d.f.=5.

* Significant at α=0.05 levels.

Due to ordinal nature of variables in Tables 2, 3 & 4, Spearman rank correlation coefficients are computed to determine their linear relationships. Results reveal that there is little or no relationship (|r| < .25) among the variables between Tables 2 & 3 (except with Physical Activities Engagement), 2 & 4, 3 & 4. However, there is a fair degree of relationship between variables in Table 2 with Physical Activities Engagement in Table 3 with coefficients ranges from 0.298 to 0.485.

Table 5. Spearman Rank Correlation Coefficients between Physical Activities Engagement and Intensity of Physical Exercies

	Vigorous physical exercise	Moderate physical exercise	Walk for at least 10 minutes at a time
**Physical Activities Engagement**	0.485 (p<.001*,n=1539)	0.298 (p<.001*, n=1555)	0.348 (p<.001*,n=1565)

*Significant at α=0.05 levels.

For peer review only

Despite the perceived low level of being physically active (45.4%) and eating healthy (34.5%), the majority of participants found that engaging in physical activity (88.7%) and eating healthy foods (74.3%) are pleasant. Furthermore, 98.6% of participants believed that being active and eating healthy are beneficial. More than half strongly agreed that engaging in physical activity (73.1%) and eating health food (66.6%) would help them maintain good health. Out of 1606, 75.9% of participants communicated that they would engage in physical activity and 75.5% said they would eat healthy foods within the next month. Many were willing to continue to be physically active for the next 6 months (81.5%) and eat healthy foods (79.2%).

Reported Health Status

Nearly half of the study participants (49.1%) reported good health, while the remaining were of excellent (26.2%), fair (19.3%), or poor (4.5%) health. Most participants did not have a medical history of heart attack, cancer, psychiatric illness, polycystic ovarian syndrome, or fractures. Overall, 10% to 13% of the participants reported high blood cholesterol level, high blood pressure, diabetes, obesity, or bowel problems (Table 6).

Table 6. Health Status of Study Participants – (n =1606)

MEDICAL HISTORY	YES N (%)	NO N (%)	P-VALUES
HIGH BLOOD PRESSURE [A]	206 (12.9)	1389 (87.1)	<.001*
BOWEL PROBLEMS [C]	195 (12.2)	1409 (87.8)	<.001*
HIGH BLOOD CHOLESTEROL [B]	183 (11.5)	1411 (88.5)	<.001*
DIABETES [D]	180 (11.2)	1422 (88.8)	<.001*
OBESITY [E]	162 (10.1)	1443 (89.9)	<.001*
ALLERGIES [F]	146 (9.1)	1457 (90.9)	<.001*
STOMACH PROBLEMS [E]	130 (8.1)	1475 (91.9)	<.001*
ASTHMA [E]	73 (4.5)	1532 (95.5)	<.001*
FRACTURES [E]	46 (2.9)	1559 (97.1)	<.001*

For peer review only

POLYCYSTIC OVARIAN SYNDROME [D]	45 (2.8)	1557 (97.2)	<.001*
OSTEOPOROSIS [G]	44 (2.8)	1553 (97.2)	<.001*
PSYCHIATRIC ILLNESS [E]	12 (0.7)	1593 (99.3)	<.001*
HEART ATTACK [C]	10 (0.6)	1594 (99.4)	<.001*
CANCER	2 (0.1)	1604 (99.9)	<.001*
STROKE [C]	1 (0.1)	1603 (99.9)	<.001*

[a] 11 participants did not answer this question, [b] 12 participants did not answer this question, [c] 2 participants did not answer this question, [d] 4 participants did not answer this question, [e] 1 participants did not answer this question, [f] 3 participants did not answer this question, [g] 9 participants did not answer this question,

All p-values are computed by one sample Chisquare test, with d.f.=1.

* Significant at α=0.05 levels

Table 7 summarizes multivariate logistic regression analyses results performed with selected independent factors that may be used as indicators to predict participant engaged in physical activities. Married had 0.68 times (p=0.020), older people had 0.65 times (p=0.002) and female had 0.684 times (p<0.001) of the odds of physical activities engagements. Highest level of education is also a statistical significant predictor of physical activities engagements with $\chi2$ (5) =11.73, p=0.039).

Table 7. Association between Selected Factors and Physical Activities engagement

**Predictors of Physical Activities Engagement**	**Adjusted OR (95% CI)**	**P value**
Marital Status		
Not married (reference)	1.0	
Married	0.68 (0.50 – 0.94)	0.020*
Agegroup		
18-39 (reference)	1.0	
40 & older	0.65 (0.49 – 0.86)	0.002*
Gender		
Male (reference)	1.0	
Female	0.38 (0.29 – 0.49)	<0.001*

For peer review only

Table 7. Association between Selected Factors and Physical Activities engagement

	Adjusted OR (95% CI)	P value
Education of Participant (Wald $\chi^2(5)=11.73$)		0.039*
≤Primary/intermediate school(reference)	1.0	
Primary/Junior High	0.69 (.19 – 2.53)	0.571
High School	0.94(.26 - 3.32)	0.920
Trade	0.22(.05 - .97)	0.045*
University	0.77(.22 - 2.70)	0.687
Other	0.88(.24 – 3.22)	0.849

Model summary		
-2 Log likelihood	Cox & Snell R Square	Nagelkerke R Square
1426.38	0.08	0.11

For peer review only

Table 8 summarizes multivariate logistic regression analyses results performed with selected independent factors that may be used as indicators to predict participant eating healthy food. Older people had 0.66 times (p=0.001) and people live in Al-Wakra (South of Qatar) had 0.66 times (p=0.019) of the odds of eating healthy food. Live area is also a statistical significant predictor of eating healthy food with $\chi2$ (2) =8.09, p=0.018.

Table 8. Association between Selected Factors and 'do you think you eat healthy food?'

Predictors of 'do you think you eat healthy food?'	Adjusted OR (95% CI)	P value
Age group		
18-39 (reference)	1.0	
40 & older	0.66 (0.51 – 0.85)	0.001*
Live Area(Wald $\chi^2(2)$=8.09)		0.018*
Al-Doha (reference)	1.0	
Al-Wakra (South of Qatar)	0.62 (0.41 – 0.92)	0.019*
Al-Khor/ Al Thakhira (North of Qatar)	1.29 (0.87 – 1.90)	0.204

Model summary		
-2 Log likelihood	Cox & Snell R Square	Nagelkerke R Square
1431.66	0.02	0.02

Discussion

In this cross-sectional community-based survey study, we examined the level of physical activity, dietary habits, and health status among adult Arabic speakers living in the State of Qatar. Our findings highlight the low physical activity levels in study participants. Studies indicated that low physical activity level in the Middle East is related to weather condition [23, 24]. The environmental factor of a hot desert climate is a barrier for people to engage in exercise and participate in outdoor activities. In summer, temperature is very high in Qatar (30–50 degree Celsius), which restricts outdoor activities such as walking, cycling,

For peer review only

and jogging. Other reported barriers include lack of interest, motivation, and information about the benefits of exercise, stress, excessive internet and computer usage, and lack of exercise facilities [15, 25].

The findings of this study indicated that there is differences in the level of vigorous and mild (i.e. casual walking) activity between male and female participants. Previous studies have reported similar results with females being less involved in regular physical activity [26, 27]. Recent work in Qatar revealed that men were more physically active than women, both for walking (74.6% of men versus 55.3% of women) and intense sports (16.5% of men versus 8.9% of women) [28]. An Omani study found that females were significantly more inactive compared to males (76.9% versus 33.3%) [29]. Direct comparisons across studies are complicated due to multiple factors such as methodological variations, ethnicity, geographical location and cultural values [30]. However, low involvement of female in regular physical activities in Qatar is possibly related to cultural and social norms as women in many Islamic countries need to be accompanied by a male family member when going outdoors, a barrier towards physical activities, and a general lack of social support for exercise [31-33]. Even thought the present study's data indicated that less female participants are physically active, older married females had slightly higher the odds of involving in physical activity. We anticipated that this might be related to the fact that older married females are more physically involved in family domestic activities.

Sitting has been highlighted as a specific marker of sedentary behaviors [34]. In this study, it was alarming that participants sat over 7 hours per week day and slightly more than half (53.2%) spent 5-10 hours sitting per week day. In contrast, recently published research reported overall median time spent in sedentary activities was 179 minutes (2.98 hours) per day among Qatari nationals [24]. More sitting time and insufficient physical activity are deleteriously and are significantly associated with higher health risk factors such as

For peer review only

abdominal obesity, dyslipidemia, hyperglycemia and hypertension among the adult population [35].

In the present study, about two thirds of the participants were either overweight or obese with mean BMI of 28.03 Kg/m^2. Our findings are in consistent with a previous study conducted in Qatar, where 70.1% of participants had a BMI equal to or above 25 kg/m^2 (classified as overweight and obese) [28]. The prevalence of obesity in GCC countries is among the highest in the world. According to the Global status report in 2014 [17], rates of obesity reached more than 37% in the UAE, almost 40% in Kuwait, and more than 42% in Qatar. Similarly, high body-mass index in Saudi Arabia (34.7%) and in Lebanon (31.9%) have been reported [36].

The rapid increase in wealth and subsequent development of Arab countries has led to changes in lifestyle. One of the many consequences of region development, secondary to abundant oil resources, has been a significant change in eating habits, with the GCC's former Mediterranean-style diet of traditional products (e.g., dates, vegetables, wheat) replaced by a reliance on fast food that is dominated by refined and processed meals heavy in sugar and other carbohydrates [37]. This might help explaining our findings which found older participants who lived outside of the country's capital had a slightly higher the odds of eating healthy because they might be less likely to adopt Western-style diet. There is strong evidence for the benefits of healthy diet in the primary prevention of major cardiovascular events, and for reducing the risk of diabetes among people with high cardiovascular risk [38, 39]. In a recent qualitative study, Donnelly and colleagues [14] examined socio-cultural factors that influenced the healthy lifestyles (i.e., diet, physical activity, non-smoking) of 50 Arabic women with heart disease living in Qatar. The participants reported that their diets tended to be high in salt, sugar, and fats. Their diets were also influenced by traditional cultural beliefs and values. For example, women were often invited to each other homes

For peer review only - http://bmjopen.bmj.com/site/about/guidelines.xhtml

where sweets and coffee were served. The women ate the foods that were offered because refusing to eat the food would be considered socially unacceptable behavior. Furthermore, Donnelly and colleagues [15] reported that social support, cultural values, religion, hot desert climate, heart disease, changing socio-demographic and economic conditions impacted both positively and negatively on the ability of these women to pursue a healthy lifestyle.

Overall, participant's dietary habits in this study were not found to be healthy: 40.6% ate white bread daily and had pasta and cakes or pastries 2-4 times per week. Non-carbohydrate-containing foods such as fruits, legumes, vegetables, minimally processed whole grains are healthy and cardio-metabolic protective, while foods rich in refined grains for instance white bread, white rice, crackers, cereals, bakery desserts, starches, and added sugars are associated with weight gain [10]. A study conducted in Oman reported few gender differences with respect to eating habits, except in dairy and meat consumption where 62.5% and 55.5% of males consumed more than 3 servings, compared to 18.78 % and 35.2% of females, respectively [29]. Dietary behaviors are mainly influences by individual factors (taste preferences, dietary knowledge, stress, body image, former eating habits and physical activity level) and environmental factors (availability and accessibility, costs of food products) [40]. Given that more female than male in the categories of underweight and obese class III in our study, facilitating Arab women's engagement in healthier lifestyle needs to be emphasized. There is a need to develop collaboration among health care professionals, academics, public health professionals, and policy makers to improve the dietary behaviors and lifestyle of the population. In the UK, the National Institute for Health and Care Excellence (NICE) recommends that primary care practitioners deliver, brief physical activity advice to lethargic adults, and follow this up at next appointments [41].

For peer review only

Conclusion

Study results concluded there is insufficient level of physical activity and poor dietary behavior. Insufficient level of physical activity and unhealthy dietary present a great health risk. There is a need to develop a nationwide health promotion program aims at increase knowledge about the benefit of being physically active and eating healthy. Awareness of and adherence to the new Arabic dietary guidelines should be encourage by health care policy makers and health care providers. The findings of this study might provide insights and information necessary for the development of public health policies and promotion programs in Qatar and in the Middle East region.

Study Limitations

The present study nonetheless has multiple limitations that should be considered when interpreting the results. Non-probability convenience sampling limits the ability to generalise the findings from this study. The findings of this study may not be valid for generalization to other population segments of different race/ethnicity and socio-economic status due to geographic/cultural differences. However, the findings are communicable to the population with similar ethnic and cultural backgrounds. In this study, participants were 18 years of aged and over. Younger participants (age <18 years) may possibly give another view of perception in regard to their physical activity and dietary behaviour. In addition, information on dietary habits in the present study was based on the frequency of consumption of food items without much consideration of quantity or portion size. Future research is needed to investigate the association between Arabs attitude, perceived behavioural control and their physical activity level.

For peer review only

Acknowledgments

This study and publications was made possible by a grant from Qatar National Research Fund under its National Priority Research Program (**NPRP 6 – 049 – 3 – 009)** for which we are very grateful. Its contents are solely the responsibility of the authors and do not necessarily represent the official views of Qatar National Research Fund.

We thank our study participants who accepted to join us and share their experiences. We hope through this report we repay them for their insight and trust. We give special thanks to the staff at universities and college, and Primary Health Care Centers who helped us to recruit the research participants, to our project coordinator, Shima Sharara and to our research assistants: Nagla HagOmer, Sara Saeed, Samira Mouhamed. We also thank Muhammad Siddiqui for his assistance with this report.

Authors' contributions

TTD: Contributed to the conception and design of the study and the acquisition, analysis, and interpretation of data, drafted the manuscript, and gave final approval of the manuscript version submitted for publication.
TF: Contributed to the conception and design of the study and the acquisition of data, revised the manuscript, and gave final approval of the manuscript version submitted for publication.
AA: Contributed to the conception and design of the study and the acquisition of data, reviewed the manuscript critically for content, and gave final approval of the manuscript version submitted for publication.

Funding

This study was made possible by a grant from the Qatar National Research Fund under its National Priorities Research Program (**NPRP 6 – 049 – 3 – 009**). Its contents are solely the responsibility of the authors and do not necessarily represent the official views of the Qatar National Research Fund.

Conflict of interest

The authors report no relationships that could be construed as a conflict of interest.

Ethics approval

The ethical approvals were obtained from several ethics review boards (IRBs) which include: Hamad Medical Corporation/Weill Cornell Institutional Review Board (IRB NO: 13-00051), the University of Calgary's Conjoint Health Research Ethics Board (REB13-0347), Qatar Primary Health Care Research Committee (Reference NO: PHCC/RC/14/02/004), Qatar University (QU-IRB 244-E/13), College of North Atlantic in Qatar (CNAQ Approval NO: 2015-3), and Qatar Supreme Council of Health (SCH-A-UCQ-050).

Data sharing statement

No additional data are available.

For peer review only

References

1. Ding D, Lawson K, Kolbe-Alexander T, Finkelstein E, Katzmarzyk P, van Mechelen W, et al. The economic burden of physical inactivity: a global analysis of major non-communicable diseases. The Lancet. 2016;388(10051):1311-24. doi: https://doi.org/10.1016/S0140-6736(16)30383-X.

2. Lee IM, Shiroma EJ, Lobelo F, Puska P, Blair SN, Katzmarzyk PT. Effect of physical inactivity on major non-communicable diseases worldwide: an analysis of burden of disease and life expectancy. The Lancet. 2012;380(9838):219-29. doi: https://doi.org/10.1016/S0140-6736(12)61031-9.

3. Yusuf S, Hawken S, Ôunpuu S, Dans T, Avezum A, Lanas F, et al. Effect of potentially modifiable risk factors associated with myocardial infarction in 52 countries (the INTERHEART study): case-control study. The Lancet. 2004;364(9438):937-52. doi: doi.org/10.1016/S0140-6736(04)17018-9.

4. Global recommendations on physical activity for health The World Health Organization website. . [cited 2012 October]. Available from: http://www.who.int/dietphysicalactivity/factsheet_recommendations/en/2011.

5. Mabry R, Reeves M, Eakin E, Owen N. Evidence of physical activity participation among men and women in the countries of the Gulf cooperation council: a review. Obes Rev. 2010;11(06):457-64. doi: doi: 10.1111/j.1467-789X.2009.00655.x.

6. Ramirez FD, Chen Y, Di Santo P, Simard T, Motazedian P, Hibbert B. Association Between Self□Reported Potentially Modifiable Cardiac Risk Factors and Perceived Need to Improve Physical Health: A Population□Based Study. Journal of the American Heart Association. 2017;6.

7. Booth FW, Roberts CK, Laye MJ. Lack of exercise is a major cause of chronic diseases. . Compr Physiol. 2012;2(2):1143–211. . doi: doi: 10.1002/cphy.c110025.

8. Collese T, Nascimento-Ferreira M, de Moraes A, Rendo-Urteaga T, Bel-Serrat S, Moreno L, et al. Role of fruits and vegetables in adolescent cardiovascular health: a systematic review. Nutr Rev. 2017;ahead of print. doi: 10.1093/nutrit/nux002.

For peer review only

9. World Health Organization (WHO) World Health Statistics—2014. World Health Organization. Available from: http://apps.who.int/iris/bitstream/10665/112738/1/9789240692671_eng.pdf?ua=1.

10. Mozaffarian D, Hao T, Rimm E, Willett W, Hu F. Changes in Diet and Lifestyle and LongTerm Weight Gain in Women and Men. N Engl J Med. 2011;364:2392-404.

11. Badran M, Laher I. Obesity in arabic-speaking countries. J Obes. 2011;2011:686430. doi: 10.1155/2011/686430. PubMed PMID: 22175002; PubMed Central PMCID: PMCPMC3228340.

12. Mehio Sibai A, Nasreddine L, Mokdad AH, Adra N, Tabet M, Hwalla N. Nutrition transition and cardiovascular disease risk factors in Middle East and North Africa countries: reviewing the evidence. Ann Nutr Metab. 2010;57(3-4):193-203. Epub 2010/11/23. doi: 10.1159/000321527. PubMed PMID: 21088386.

13. Ng SW, Zaghloul S, Ali H, Harrison G, Yeatts K, El Sadig M, et al. Nutrition transition in the United Arab Emirates. Eur J Clin Nutr. 2011;65(12):1328-37. Epub 2011/07/21. doi: 10.1038/ejcn.2011.135. PubMed PMID: 21772317; PubMed Central PMCID: PMCPMC3304306.

14. Donnelly TT, Al Suwaidi J, Al Bulushi A, Al Enazi N, Yassin K, Rehman AM, et al. The influence of cultural and social factors on healthy lifestyle of Arabic women. Avicenna. 2011;(2011):3. doi: 10.5339/avi.2011.3.

15. Donnelly TT, Al Suwaidi J, Al Bulushi A, Al Enazi N, Yassin K, Rehman AM, et al. Qatari women living with cardiovascular diseases-challenges and opportunities to engage in healthy lifestyles. Health Care Women Int. 2012;33(12):1114-34. doi: 10.1080/07399332.2012.712172. PubMed PMID: 23153347.

16. World Health Organisation: Obesity and overweight 2016. Available from: http://www.who.int/mediacentre/factsheets/fs311/en/.

17. Global status report on noncommunicable diseases. Geneva: World Health Organization. 2014. Available from: http://apps.who.int/iris/bitstream/10665/148114/1/9789241564854_eng.pdf?ua=1.

For peer review only

18. Qatar Statistics Authority, Ministry of Development Planning and Statistics, Population and Social Statistics. 2016.

19. Institute of Chartered Accountants in England and Wales (ICAEW) Economic Insight: Middle East. Quarterly briefing Q3. 2016. Available from: http://www.icaew.com/-/media/corporate/files/technical/economy/economic-insight/middle-east/me-q3-2016-web.ashx

20. WG C. Sampling Technique. 3rd ed ed: John Wiley & Sons: New York; 1997.

21. Craig C, Marshall A, Sjöström M, Bauman A, Booth M, Ainsworth B, et al. International physical activity questionnaire: 12-country reliability and validity. Med Sci Sports Excer. 2003;35(8):1381-95.

22. IPAQ scoring protocol. . Available from: https://sites.google.com/site/theipaq/scoring-protocol.

23. Ali HI BL, Bernsen RM. Barriers and facilitators of weight management: perspectives of Arab women at risk for type 2 diabetes. Health Soc Care Community. 2010;18(2):219-28. doi: doi: 10.1111/j.1365-2524.2009.00896.x.

24. Kahan D. Arab American college students' physical activity and body composition: reconciling Middle East-West differences using the socioecological model. Res Q Exerc Sport 2011;82(1):118-28.

25. Benjamin K, Donnelly T. Barriers and facilitators influencing the physical activity of Arabic adults: A literature review. Avicenna. 2013;2013(1):8. doi: 10.5339/avi.2013.8.

26. Al-Hazzaa HM, Al-Sobayel HI, Abahussain NA, Qahwaji DM, Alahmadi MA, Musaiger AO. Association of dietary habits with levels of physical activity and screen time among adolescents living in Saudi Arabia. J Hum Nutr Diet. 2014;27 Suppl 2:204-13. doi: 10.1111/jhn.12147. PubMed PMID: 23889093.

27. Fernandes RA, Christofaro DF, Casonatto JF, Kawaguti SS, Ronque ERV, Cardoso JR, et al. Cross-sectional association between healthy and unhealthy food habits and leisure

For peer review only

physical activity in adolescents. J Pediatr (Rio J). 2011;87(3):252-6. doi: doi:10.2223/JPED.2093.

28. Al-Thani MH A-TA, Al-Chetachi WF, Khalifa SAH, Akram H, Poovelil BV, Almalki BA, Bakri AH, Arora P, Badawi A. Dietary and Nutritional Factors Influencing Obesity in Qatari Adults and the Modifying Effect of Physical Activity. J Obes Weight-Loss Medic. 2015;1(1):1-7.

29. Kilani H, Al-Hazzaa H, Waly M, Musaiger A. Lifestyle Habits: Diet, physical activity and sleep duration among Omani adolescents. Sultan Qaboos Univ Med J. 2013;13(4):510–9.

30. Hamer M, Brunner E, Bell J, Batty G, Shipley M, Akbaraly T, et al. Physical activity patterns over 10 years in relation to body mass index and waist circumference: the Whitehall II cohort study. Obesity. 2013;21(12):E755-61.

31. Amin TT, Suleman W, Ali A, Gamal A, A. AW. Pattern, prevalence, and perceived personal barriers toward physical activity among adult Saudis in Al-Hassa, KSA. J Phys Act Health. 2011;8(6):775-84.

32. Qahoush R, Stotts N, Alawneh M, Froelicher E. Physical activity in Arab women in Southern California. Eur J Cardiovasc Nurs. 2010;9(4):263-71. doi: doi: 10.1016/j.ejcnurse.2010.03.002.

33. Shuval K, Weissblueth E, Araida A, Brezis M, Faridi Z, Ali A, et al. The role of culture, environment, and religion in the promotion of physical activity among Arab Israelis. Prev Chronic Dis. 2008;5(3):A88. doi: D - NLM: PMC2483551 EDAT- 2008/06/19 09:00 MHDA- 2008/08/08 09:00 CRDT- 2008/06/19 09:00 AID - A88 [pii] PST - ppublish.

34. Owen N, Bauman A, Brown W. Too much sitting: a novel and important predictor of chronic disease risk? Br J Sports Med. 2009;43(2):81–3.

35. Chu AHY, Moy FM. Joint Association of Sitting Time and Physical Activity with Metabolic Risk Factors among Middle-Aged Malays in a Developing Country: A Cross-Sectional Study. Bayer A, ed. . PLoS ONE 2013;8(4):e61723. doi: doi:10.1371/journal.pone.0061723. .

For peer review only

36. Confronting obesity in the Middle East cultural, social and policy challenges. The Economist Intelligence Unit Limited 2016. Available from: https://www.eiuperspectives.economist.com/sites/default/files/ConfrontingobesityintheMiddleEast_0.pdf

37. Gerber M, Hoffman R. The Mediterranean diet: health, science and society. . Br J Nutr. 2015;113(2):S4-10.

38. Estruch R, Ros E, Salas-Salvad J, Covas M, Corella D, Arós F, et al. Primary prevention of cardiovascular disease with a Mediterranean diet. N Engl J Med. 2013;368(14):1279-90. doi: doi: 10.1056/NEJMoa1200303.

39. Salas-Salvadó J, Bulló M, Estruch R, Ros E, Covas M, Ibarrola-Jurado N, et al. Prevention of diabetes with Mediterranean diets: a subgroup analysis of a randomized trial. Ann Intern Med. 2014;160(1):1-10. doi: doi: 10.7326/M13-1725.

40. Deliens T, Clarys P, De BI, Deforche B. Determinants of eating behaviour in university students: a qualitative study using focus group discussions. BMC Public Health. 2014;14(1):53.

41. National Institute for Health and Care Excellence. Physical activity: brief advice for adults in primary care. NICE Public Health Guidance 44. National Institute for Health and Care Excellence. 2013. Available from: https://www.nice.org.uk/guidance/ph44.

Figure 1. Gulf coast of the Arabian Peninsula

Figure 2: Distribution of BMI among participants

For peer review only

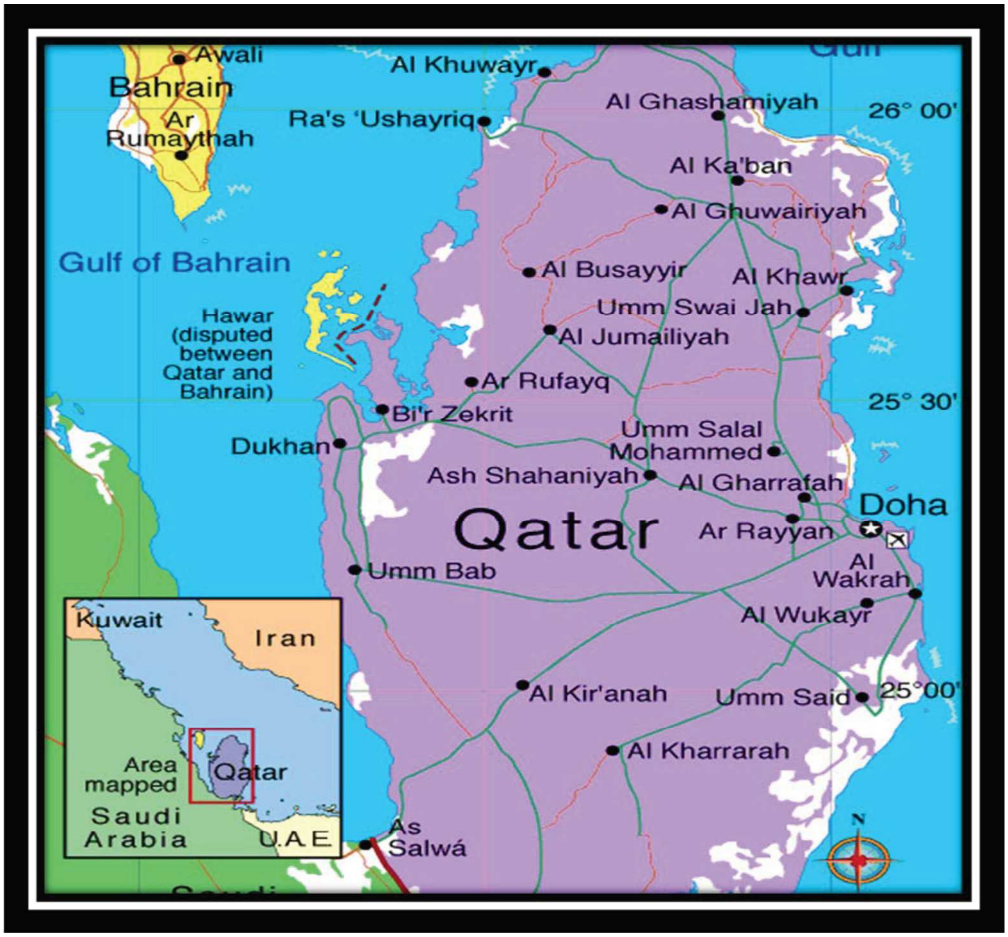

Figure 1. Gulf coast of the Arabian Peninsula

147x136mm (300 x 300 DPI)

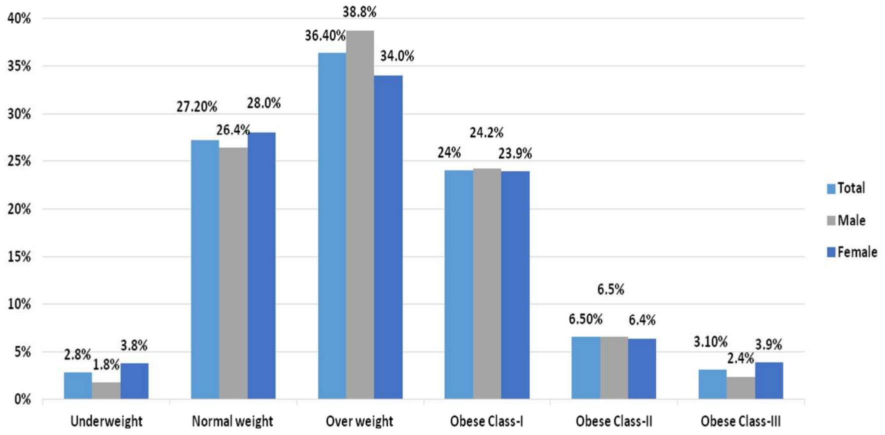

Figure 2: Distribution of BMI among participants

152x83mm (300 x 300 DPI)

BMJ Open

Fostering active living and healthy eating through understanding physical activity and dietary behaviours of Arabic-speaking adults – a cross-sectional study from the Middle East

Journal:	*BMJ Open*
Manuscript ID	bmjopen-2017-019980.R2
Article Type:	Research
Date Submitted by the Author:	02-Feb-2018
Complete List of Authors:	Donnelly, Tam Truong; University of Calgary, Nursing/Medicine<br>Fung, Tak; University of Calgary,<br>Al-Thani, Al-Anoud ; Ministry of Public Health
**Primary Subject Heading**:	Global health
Secondary Subject Heading:	Public health, Health services research, Sports and exercise medicine, Nutrition and metabolism
Keywords:	Physical activity, Dietary behaviour, Healthy lifestyle, Arabic-speaking adults, Qatar, Middle East

SCHOLARONE™ Manuscripts

For peer review only

Fostering active living and healthy eating through understanding physical activity and dietary behaviours of Arabic-speaking adults – a cross-sectional study from the Middle East

Authors:

Tam Truong Donnelly, Tak Fung, Al-Anoud bint Mohammad Al-Thani

Institutions:

Tam Truong Donnelly, PhD
University of Calgary
Faculty of Nursing and Medicine
2500 University Dr. NW
Calgary, Alberta, Canada
T2N 1N4

Tak Fung, PhD
University of Calgary
Research Computing Services
Information Technologies
2500 University Dr. NW
Calgary, Alberta, Canada
T2N 1N4

Al-Anoud bint Mohammad Al-Thani, MD
Health Promotion & Non Communication Diseases
Public Health Department
P.O. Box 42
Doha, Qatar

Corresponding author:

Tam Truong Donnelly, PhD.
University of Calgary
Faculty of Nursing and Medicine
PF 2234, 2500 University Drive NW Calgary. Alberta. Canada T2N 1N4
Tel: 1-403-220-6867
Fax: 1-403-284-4803
E-mail: tdonnell@ucalgary.ca

For peer review only

Abstract

Objectives

Physical inactivity and unhealthy diets increase the risk for diabetes, cardiovascular diseases, and cancer. Many people in Qatar are sedentary and consume diets high in fats, salt and sugar. The purpose of this study was to determine physical activity levels, food habits, and understand the variables that might predict physical activity and healthy eating behaviours among Arabic speaking adults living in the State of Qatar.

Setting

A cross-sectional community-based survey was conducted with 1606 Arab adults ≥18 years of age from March 2013 to June 2015. Using a non-probability sampling technique, participants were recruited from three universities and five primary healthcare centers in Qatar. Participants were interviewed using a structured survey questionnaire. The survey included questions regarding demography, clinical characteristics, and the participant's daily dietary practice. Physical activity level was assessed by the Arabic version of the International Physical Activity Questionnaire. Statistical analysis was performed using SPSS version 22.0.

Results

Of 1606 participants, 50.1% were male and 49.9% were female. The participants' mean (SD) body mass index was 28.03(5.85) Kg/m^2. Two thirds of the participants were either overweight (36.4%) or obese (33.6%). Within the 7 days prior to completing the questionnaire, 64% and 39.9% of study participants did not engage in vigorous or moderate physical activity, respectively. Within the 7 days prior to completing the questionnaire, the mean (SD) time for vigorous physical activity was 31.12 (59.28) minutes, 46.87 (63.01) minutes for moderate physical activity, and 42.01 (47.04) minutes for walking. One third of the participants consumed fresh fruits and vegetables once or more daily, and fish, beef, or chicken 2 to 4 times weekly. One fourth of the participants ate pasta, cakes, or pastries 2 to 4 times weekly, and 40.6 % of them ate white bread daily.

Conclusions

Participants exhibited insufficient physical activity and poor dietary habits. There is a need for a nationwide health promotion program to promote a healthier lifestyle. The information from this study can inform public health policies, programs and services in Qatar and other Middle Eastern countries.

Keywords

Physical activity, Dietary behaviour, Healthy lifestyle, Arabic-speaking adults, Qatar, Middle East

For peer review only

Article summary:

Strengths and Limitations:

- Large sample size (n=1606) that is representative of the Arab Qatari population and face-to-face interviews with all participants are major strengths of this study.
- Non-probability convenience sampling limits the generalizability of the study findings.
- This study only reports data pertaining to Arabic-speaking adults. Younger participants (age < 18) might give different perspectives on their physical activity and dietary behaviour.

For peer review only

Introduction

Physical inactivity and unhealthy diets are major health and economic problems worldwide, and are important modifiable risk factors for non-communicable diseases such as type 2 diabetes, cardiovascular disease, and some cancers [1-3]. In a recent study, nine modifiable risk factors that accounted for more than 90% of the risk for acute myocardial infarction were identified as: sedentary lifestyle, history of hypertension, diabetes, smoking, abdominal obesity, occasional-non daily consumption of fruits and vegetables, psychosocial factors, regular alcohol consumption, and a raised apolipoprotein B/ApoA1 ratio [4]. These risk factors were found to be associated with myocardial infarction irrespective of gender and age. Similarly, in the Arabian Gulf countries, the most eminent risk factors of non-communicable diseases were known as inadequate intake of fruit and vegetables, being overweight or obese, physical inactivity, high blood pressure, high blood cholesterol, and tobacco use [5]. In 2013, the cost of physical inactivity for health care systems was $53.8 billion worldwide [2]. In Qatar, the annual direct and indirect costs attributable to physical inactivity were estimated as $60.7 million in 2013 [1]. Furthermore, it costs people's lives, well-being, quality of life, and health of their families and caregivers.

According to the World Health Organization's (WHO) [6] guidelines for physical activity, healthy adults 18-64 years of age should accumulate at least 150 minutes of moderate intensity aerobic activity per week, or 75 minutes of vigorous intensity, or an equivalent combination of moderate and vigorous-intensity aerobic activity in bouts of 10 minutes or more. Similarly, the State of Qatar National Physical Activity Guidelines recommends that healthy adults 18-64 years of age should do 5 days or more of 30 to 60 minutes of moderate exercise per week or 3 days or more of 20 to 60 minutes of vigorous exercise per week [7]. However, the results of a study in the Gulf Cooperation Council countries showed that only 39.0% to 42.1% of men and 26.3% to 28.4% of women were

For peer review only

physically active for at least 150 minutes per week [8]. According to the Qatar STEPwise survey conducted in 2012, 45.9% of the participants were engaged in low levels of activity per week, and 31.3% were engaged in high levels of activity per week. The median time spent in physical activity on an average day was 37 minutes. Of all the study participants, 71.3% were not engaged in vigorous activity [9]. According to WHO, the highest prevalence of insufficient physical activity (31%) was located in the Eastern Mediterranean Region. In Qatar, the prevalence of insufficient physical activity in adults was reported as 41.6% [10]. Ramirez et al. [11] observed that 1 in 5 patients with the highest cardiac risk did not perceive the need to improve their physical health.

According to a recent systematic review, regular intake of fruits and vegetables was inversely associated with high systolic blood pressure, abdominal obesity, triglycerides, high-density lipoprotein cholesterol, and metabolic syndrome [12]. Along with WHO healthy diet guidelines, Qatar's Food-based Dietary Guidelines recommend that people should eat healthy choices from different food groups, limit sugar, salt and fat, and maintain a healthy weight [13]. However, urbanization alongside the modernization and westernization in Arab countries have shaped dietary habits; people in Qatar have ready access to foods high in sugar, fats, and salts (e.g., fast food, carbonated beverages) [14-16]. According to the Qatar STEPwise approach to chronic disease risk factor surveillance, adult participants 18-64 years of age consumed 0.8 servings of fruit and 1.4 servings of vegetables on average per day, and 91% ate less than five servings of fruit and/or vegetables on average per day [9]. Situated on the Gulf Coast of the Arabian Peninsula (Figure 1), with a population of nearly 2.6 million [17], the State of Qatar had the fastest growing economy in the Gulf region and the highest gross domestic product per capita due to its abundant oil and natural gas revenues [18]. This steep economic growth over the decades seemed to influence availability of food and patterns

For peer review only

of food consumption. However, the current literature on dietary practice of the Arab Qatari population is scarce.

Given that obesity is linked to physical inactivity and/or unhealthy diet and that the fundamental cause of obesity and overweight is an energy imbalance between calories consumed and calories burned [19], lifestyle changes such as increased physical activity, decreased sedentary lifestyle, and healthy dietary changes are necessary for weight management [20]. According to WHO [19], more than 1.9 billion adults worldwide were overweight in 2014, of those, over 600 million adults were obese. Currently, there is limited information on the level of physical activity and dietary habits among adults in Qatar. The main purpose of this study was to determine the current physical activity levels and dietary habits, and understand the variables that might predict physical activity and healthy eating behaviours among Arabic speaking adults living in the State of Qatar.

Figure 1. Gulf Coast of the Arabian Peninsula

Methods

Participants

A cross-sectional, community-based survey with Arabic speaking adults living in Qatar was conducted from March 2013 to June 2015. Participants were eligible for inclusion in the study if they were (1) 18 years or older, (2) self-identified as an Arabic-speaker, (3) born and/or raised in Qatar, or lived in Qatar for at least five years, and (4) willing to commit a minimum of 60 minutes for the questionnaire. We approached and recruited participants at designated data collection sites – three universities and five health care centers in the capital Doha and areas south and north of Doha in Qatar, to ensure diversity of participants that can closely represent the general Arab population in Qatar. Although random selection helps to reduce selection biases, this sampling technique was not feasible for this research. It is difficult to gain access to Arab populations because of sociocultural beliefs and practices that

For peer review only

value privacy. Hence, we used a purposeful, non-probability convenient sampling technique. We realized that this increases the risk of selection biases and limits the generalizability of the research findings. To help offset these limitations, we recruited and randomly selected participants at different times of the days, weeks, or months of the year at the designated data collection sites.

Trained interviewers who are fluent in both Arabic and English identified eligible participants based on the inclusion criteria. Providing the eligible individual answered “yes” to the screening questions and wished to continue, the interviewer: (1) provided the participant with a short explanation of the study, (2) advised the participant that his/her participation was strictly voluntary, (3) advised him/her that measures would be taken to help ensure confidentiality, and (4) answered any of the participant’s questions. When the participant agreed to participate, this was considered consent by assent. The interviewer then enrolled the participant in the study and administered the survey. Recruitment continued until the determined sample size was reached.

According to the Qatar census (2010) data, there were 1,008,937 female and male (843,441 male and 165,496 female) between the ages of 15 to 75+ living in Doha, Al Wakra (South of Qatar), Al Khor and Al Thakhira (North of Qatar). Based on the Cochran’s formula using a margin of error of 3.5% (95% confidence interval), a sample size of 781 female and 784 male was determined to be a representative sample of the Arabic speaking adults living in the above four regions [21]. Please note that we did not have access to census data that included individuals aged 18 and older. The census data we used includes ages of 15+.

Ethical approvals were obtained from the Hamad Medical Corporation/Weill Cornell Institutional Review Board (IRB NO: 13-00051), the University of Calgary~~’s~~ Conjoint Health Research Ethics Board (REB13-0347), Qatar Primary Health Care Research Committee (Reference NO: PHCC/RC/14/02/004), Qatar University (QU-IRB 244-E/13), College of

For peer review only

North Atlantic in Qatar (CNAQ Approval NO: 2015-3), and Qatar Supreme Council of Health (SCH-A-UCQ-050). We acknowledged that participants might not be familiar with the research process because little research has been conducted in Qatar, thus, the research team members carefully explained the project to participants. To enhance accessibility, all project information was available in both Arabic and English. Prior to the start of the study, an introductory letter was sent to the university and community health clinic sites. Formal and informal presentations were provided to all staff at each site at the beginning of the project. Written informed consent was waived, but verbal consent to participate in the study was obtained from each participant. All participants were assured that information would be confidential. No incentive was given to the participants.

Data Collection

Data collection was carried out using a structured survey questionnaire designed for this study. The survey instrument was developed based on literature reviewed. The survey questionnaire has four sections. The first section consists of questions asking for demographic information of the participants. The second section contains questions regarding levels of physical activity using the International Physical Activity questionnaire (IPAQ) [22]. The third section pertains to the participant's current and past history of chronic diseases. The last section consists of questions that can assess the participant's daily food intake.

All of the study material and questions were translated to Arabic and back translated to English to ensure lexical equivalence. To ensure the questionnaire items were relevant to the context in Qatar, we conducted a pilot study using six focus groups stratified by age (18-30 years, 31-50 years, 51+ years) and sex (male, female). We conducted separate focus groups for male and female to be culturally appropriate and stratified by age to group people who were likely to have similar life experiences. Following the focus groups, the

For peer review only

questionnaire was further pilot tested with 12 males and 12 females recruited from urban and semi-urban settings who represented different age groups. Based on the pilot test, we further refined the data collection protocol and the questionnaire. Experienced and specially trained bi-lingual (Arabic and English) research assistants conducted structured interviews in Arabic.

Body Mass Index. Obesity status was determined by the Body Mass Index (BMI). BMI represents the standard method used by both the WHO and the governmental health sectors of the majority of nations to determine whether a person is obese or overweight. BMI is determined by dividing the individual's weight measured in kilograms by his/her height measured in meters squared. Obesity and overweight were classified based on BMI as: Underweight ≤ 18.5 kg/m^2, Normal Weight 18.5 – 24.9 kg/m^2, Overweight 25 – 29.9 kg/m^2, Obese Class-I 30 – 34.9 kg/m^2, Obese Class-II 35 – 39.9 kg/m^2, Obese Class-III ≥ 40 kg/m^2 [23].

Physical Activity. IPAQ asks participants to recall their physical activities in the last 7 days. The results allow researchers to classify participants into one of the three levels of physical activity (i.e., low, moderate, or high). IPAQ has evidence for reliability and validity for monitoring levels of physical activity among adults 18-65 years in diverse settings [22, 23]. For this study, we used the short telephone version, which was translated into Arabic. The IPAQ website provides protocols related to data cleaning, processing, scoring, and translation [23].

Dietary Behavior. Dietary behavior was explored based on data collected using the study questionnaire which provides details about daily or weekly frequency of consumption for various food groups including fruits, vegetables, proteins (fish, beef, lamb or chicken), carbohydrates (bread, cereals, or pasta), milk products and other food items (soft drinks, juices or nuts) consumption, including number of servings per day. For example, questions were asked: How often do you eat fresh fruits/ green leafy vegetables/ fish/ cakes and

For peer review only

pastries, etc.? Participants could respond: Never; Seldom; Once a week; 2-4 times a week; 5-6 times a week; Once or more daily; Don't know.

Health status was reviewed based on data collected using the study questionnaire. The questionnaire included questions regarding history of chronic diseases such as hypertension, diabetes, dyslipidemia, stroke, obesity, history of fractures, and allergies.

Data Analysis

Data analyses were conducted by a senior biostatistician using SPSS (IBM SPSS Statistics 22.0). Descriptive statistics analyses (frequencies, means, and standard deviations (SD) for interval variables, frequency, and proportion for categorical variables) were performed for the study variables. Data were expressed in frequencies for questionnaire responses calculated for all variables in numbers and percentages. The Mann-Whitney test was used to compare differences between two groups with ordinal data, Chi -Square test was used to compare difference between two groups with categorical data, and an independent sample t-test was used to compare group differences with interval data. Multivariate logistic regression analysis was performed to identify the variables that predict physical activity (PA), healthy diet (HD) engagement, and IPAQ group. Independent variables, such as living area, marital status, nationality, age group, education, health status, household income and sex were selected using the criteria for the method of forward stepwise (Wald $\chi 2$, pin=0.05,pout=0.10). The equation used to build the model is: $\mathbf{\ln(p/(1-p))=\alpha_{0+}\alpha_1 x_1+ \alpha_2 x_2+\ldots..\alpha k x k}$, where p=probability of PA, HD engagement and IPAQ group respectively, $\mathbf{x_1,x_2,\ldots\ldots\ x_k}$ are significant predictors after forward stepwise logistic regression procedure. Statistical significance levels were established at alpha=0.05.

For peer review only - http://bmjopen.bmj.com/site/about/guidelines.xhtml

Results

Participants Characteristics

We approached 3081 participants, of which 1606 participants who met the study's inclusion criteria participated in the study (response rate of 52.1%). Eight hundred and four (50.1%) were male and 802 (49.9%) were female. The majority of study participants were under the age of 60 years. Approximately 20.8% of the participants belonged to the age group of 40-49 years. Almost all participants were Muslim (99.1%). More than half of the study participants were married (59.7%), and 40.3% were without a spouse. A total of 30.3% of the study's participants were Qatari nationals. Close to half of the participants (43.8%) came from North African countries (Egypt, Libya, Tunisia, Algeria, Morocco, and Mauritania). The citizens of the Levant countries (Syria, Lebanon, Palestine, and Jordan) constituted 19.9%. The rest of participants were from the Gulf Cooperation Council (GCC) and other countries. Overall, more than one-third of the participants completed university (37.8%), and 42% completed high school. Nearly one third of the participants' (32.7%) household income ranged between US $2746 and $5491 (QAR 10,000-19,999) per month. Most participants were nonsmokers (79.1%), 77.9% of the participants resided in Doha, 9.4% lived in Al Wakra, and 12.6% were residents of Al Khor (Table 1).

For peer review only

Table 1. Selected Demographic Characteristics of Participants (n = 1606)

Characteristics	Total n (%)	Male n (%)	Female n (%)	P-values
Age (years)[a]				
18-29	517 (32.3)	261 (32.6)	256 (32)	
30-39	557 (34.8)	258 (32.3)	299 (37.4)	
40-49	333 (20.8)	145 (18.1)	188 (23.5)	

50-59	136 (8.5)	90 (11.3)	46 (5.8)	
60-69	49 (3.1)	39 (4.9)	10 (1.3)	
70+	8 (0.5)	7 (0.9)	1 (0.1)	<.001*
Marital Status				
Single/never married	595 (37)	307 (38.2)	288 (35.9)	
Married	958 (59.7)	487 (60.6)	471 (58.7)	<.001*
Separated/Divorced/Widowed	53 (3.3)	10 (1.2)	43 (5.4)	
Nationality				
Qatari	487 (30.3)	190 (23.6)	297 (37.1)	
North Africa	704 (43.8)	415 (51.7)	289 (36)	
Levant	320 (19.9)	159 (19.7)	161 (20.1)	
Other GCC	33 (2)	12 (1.5)	21 (2.6)	
Other	61 (3.8)	28 (3.4)	33 (4.2)	<.001*
Level of Education[b]				
Never went to school	18 (1.1)	8 (1)	10 (1.2)	
Primary school	115 (7.2)	45 (5.6)	70 (8.7)	
High school	685 (42)	330 (41.1)	355 (44.3)	
Trade school	34 (2.1)	26 (3.2)	8 (1)	
University	607(37.8)	319 (39.7)	288 (36)	
Other	145 (9)	75 (9.3)	70 (8.7)	0.003*
Monthly Income in USD[c]				
les that $274	7 (0.6)	5 (0.8)	2 (0.3)	
$275- $2745	192 (15.7)	103 (16.9)	89 (14.5)	
$2746- $5491	401 (32.7)	224 (36.7)	177 (28.8)	
$5492- $8237	292 (23.8)	140 (23)	152 (24.7)	
$8238- $10983	133 (10.9)	57 (9.3)	76 (12.4)	
$10984- $13729	73 (6)	26 (4.3)	47 (7.6)	
$13730 - $16476	49 (4)	22 (3.6)	27 (4.4)	
more than $16476	78 (6.4)	33 (5.4)	45 (7.3)	0.007*
Smoking[d]				
Yes	334 (20.9)	293 (36.8)	41 (5.1)	
No	1263 (79.1)	504 (63.2)	759 (94.9)	<.001*
Living Area				
Al-Doha	1251 (77.9)	626 (77.9)	625 (77.9)	
Al-Wakra	151 (9.4)	78 (9.7)	73 (9.1)	
Al-Khor	202 (12.6)	98 (12.2)	104 (13)	

Al Thakhira	2 (0.1)	2 (0.2)	0 (0)	0.505

[a] 6 participant did not answer this question, [b] 2 participant did not answer this question,
[c] 381 participant did not answer this question, [d] 9 participant did not answer this question.
*Significant at α=0.05 levels

Four MET-minutes/week (Walking, Moderate, Vigorous and Total) are calculated for each participant. Based on the IPAQ criteria [23], each participant is classified into one of the three physical activity categories: Low, Moderate and High. IPAQ group: Low (n=348, 21.7%), Moderate (n=854, 53.2%), High (n=390, 24.3%). Fourteen participants (0.9%) could not be classified into any of the IPAQ groups due to missing values. The mean (SD) of Walking, Moderate, Vigorous and Total MET-minutes/week were 562.12 (791.31), 745.03 (1330.51), 809.60 (1803.51) and 2062.80 (2617.40) respectively (Table 2).

Table 2. Relationship between Selected Demographic Characteristics of Participants, Health Status and Medical History with Physical Activity Category (Low, Moderate, High)

Characteristics	Total n (%)	Low n (%)	Moderate n (%)	High n (%)	P-values
Age (years)					
18-29	507 (32.0)	95 (27.5)	218 (25.6)	194 (49.7)	
30-39	556 (35.1)	120 (34.8)	327 (38.4)	109 (27.9)	
40-49	330 (20.8)	78 (22.6)	201 (23.6)	51 (13.1)	
50-59	136 (8.6)	40 (11.6)	70 (8.2)	26 (6.7)	
60-69	49 (3.1)	10 (2.9)	29 (3.4)	10 (2.6)	
70+	8 (0.5)	2 (0.6)	6 (0.7)	0 (0.0)	<.001*
Marital Status					
Single/never married	586 (36.8)	117 (33.6)	274 (32.1)	195 (50.0)	
Married	955 (60.0)	216 (62.1)	551 (64.5)	188 (48.2)	
Separated/Divorced/Widowed	51 (3.2)	15 (4.3)	29 (3.4)	7 (1.8)	<.001*

For peer review only - http://bmjopen.bmj.com/site/about/guidelines.xhtml

Nationality					
Qatari	482 (30.3)	131 (37.6)	244 (28.6)	107 (27.4)	
North Africa	698 (43.9)	131 (37.6)	388 (45.5)	179 (45.9)	
Levant	318 (20.0)	65 (18.7)	181 (21.2)	72 (18.5)	
Other GCC	33 (2.1)	7 (2.0)	14 (1.6)	12 (3.1)	
Other	60 (3.8)	14 (4.0)	26 (3.0)	20 (5.1)	0.014*
Level of Education					
Never went to school	18 (1.1)	3 (0.9)	9 (1.1)	6 (1.5)	
Primary school	115 (7.2)	30 (8.6)	59 (6.9)	26 (6.7)	
High school	676 (42.5)	135 (38.9)	319 (37.4)	222 (56.9)	
Trade school	34 (2.1)	10 (2.9)	16 (1.9)	8 (2.1)	
University	602 (37.9)	140 (40.3)	371 (43.5)	91 (23.3)	
Other	145 (9.1)	29 (8.4)	79 (9.3)	37 (9.5)	<.001*
Monthly Income in USD					
les that $274	7 (0.6)	3 (1.2)	3 (0.4)	1 (0.3)	
$275- $2745	192 (15.7)	31 (12.3)	93 (13.8)	68 (23.2)	
$2746- $5491	397 (32.5)	81 (32.1)	221 (32.7)	95 (32.4)	
$5492- $8237	291 (23.9)	62 (24.6)	175 (25.9)	54 (18.4)	
$8238- $10983	133 (10.9)	28 (11.1)	77 (11.4)	28 (9.6)	
$10984- $13729	73 (6.0)	25 (9.9)	37 (5.5)	11 (3.8)	
$13730 - $16476	49 (4.0)	6 (2.4)	28 (4.1)	15 (5.1)	
more than $16476	78 (6.4)	16 (6.3)	41 (6.1)	21 (7.2)	.002*
Smoking					
Yes	332 (21.0)	67 (19.5)	183 (21.6)	82 (21.0)	
No	1251 (79.0)	277 (80.5)	666 (78.4)	308 (79.0)	0.727
Living Area					
Al-Doha	1239	279	630	330	

	(77.8)	(80.2)	(73.8)	(84.6)	
Al-Wakra	149 (9.4)	30 (8.6)	98 (11.5)	21 (5.4)	
Al-Khor	202 (12.7)	38 (10.9)	126 (14.8)	38 (9.7)	
Al Thakhira	2 (0.1)	1 (0.3)	0 (0)	1 (0.3)	0.001*
Health Status					
Fair	308 (20.5)	84 (26.2)	149 (18.5)	75 (19.8)	
Good	780 (51.8)	173 (53.9)	429 (53.2)	178 (47.1)	
Excellent	417 (27.7)	64 (19.9)	228 (28.3)	125 (33.1)	.001*
Hypertension					
Yes	205 (13.0)	64 (18.7)	107 (12.6)	34 (8.8)	
No	1376 (87.0)	278 (81.3)	744 (87.4)	354 (91.2)	<.001*
High blood cholesterol					
Yes	183 (11.6)	53 (15.3)	95 (11.2)	35 (9.1)	
No	1397 (88.4)	293 (84.7)	754 (88.8)	350 (90.9)	0.028*
Heart Attack					
Yes	10 (0.6)	2 (0.6)	8 (0.9)	0 (0.0)	
No	1580 (99.4)	346 (99.4)	846 (99.1)	388 (100.0)	0.152
Stroke					
Yes	1 (0.1)	0 (0.0)	0 (0.0)	1 (0.3)	
No	1589 (99.9)	348 (100.0)	853 (100.0)	388 (99.7)	0.213
Cancer					
Yes	2 (0.1)	2 (0.6)	0 (0.0)	0 (0.0)	
No	1590 (99.9)	346 (99.4)	854 (100.0)	390 (100.0)	0.048*
Diabetes					
Yes	179 (11.3)	45 (13.0)	99 (11.6)	35 (9.0)	
No	1409 (88.7)	301 (87.0)	754 (88.4)	354 (91.0)	0.213

Stomach problems					
Yes	127 (8.0)	29 (8.3)	74 (8.7)	24 (6.2)	
No	1464 (92.0)	319 (91.7)	780 (91.3)	365 (93.8)	0.310
Bowel problems					
Yes	192 (12.1)	42 (12.1)	104 (12.2)	46 (11.8)	
No	1398 (87.9)	304 (87.9)	750 (87.8)	344 (88.2)	0.981
Allergies					
Yes	145 (9.1)	25 (8.1)	73 (8.5)	44 (11.3)	
No	1444 (90.9)	319 (91.9)	781 (91.5)	344 (88.7)	0.212
Osteoporosis					
Yes	44 (2.8)	5 (1.4)	24 (2.8)	15 (3.9)	
No	1540 (97.2)	341 (98.6)	826 (97.2)	373 (96.1)	0.136
Fractures					
Yes	46 (2.9)	8 (2.3)	19 (2.2)	19 (4.9)	
No	1545 (97.1)	340 (97.7)	834 (97.8)	371 (95.1)	0.027*
Psychiatric illness					
Yes	12 (0.8)	5 (1.4)	5 (0.6)	2 (0.5)	
No	1579 (99.2)	342 (98.6)	849 (99.4)	388 (99.5)	0.245
Polycystic ovarian syndrome					
Yes	45 (2.8)	9 (2.6)	25 (2.9)	11 (2.8)	
No	1543 (97.2)	339 (97.4)	826 (97.1)	378 (97.2)	0.946

*significant at α=0.05 levels.

Participants' Body Mass Index

The study's research assistant measured each participant height and weight and calculated the participant's BMI according to the guideline. The mean (SD) BMI of the study participants was 28.03 (5.85) kg/m^2 and ranged from 12.8 kg/m^2 to 65.44 kg/m^2. Overall prevalence rates of underweight, normal weight and overweight were 44 (2.8%), 433 (27.2%), and 580 (36.4%) respectively. Obesity class I, II and III were observed in 383 (24%), 103 (6.5%), and 50 (3.1%) respectively (Fig 2).

Figure 2. Distribution of BMI among participants

Nearly one third of the participants were obese. The majority of obese participants were classified as Obese Class I (24.2% male and 23.9% female). Thirty-four per cent of the female participants and 38.8% of the male participants were overweight. It is important to note that more females than males were in the underweight category and obese class III category based on their BMI.

Level of Physical Activity

Sixty four percent of study participants did not perform any vigorous physical activity, and 39.9% did not perform any moderate physical activity during 7 days prior to the data collection (Table 3). Within those 7 days, participants spent a mean (SD) of 31.12 (59.28) minutes doing vigorous physical activity, 46.87 (63.01) minutes doing moderate physical activity, and 42.01 (47.04) minutes walking. Male participants spent significantly ($p < .001$) more time doing vigorous physical activity (mean rank score 859) and walking (mean rank score 831.1) compared to females (mean rank score 690.6, mean rank score 730.5, respectively). However, males (mean rank score 769.5) and females (mean rank score 789.5) spent a similar amount of time doing moderate physical activity ($p = 0.361$). In this study, participants spent a mean (SD) of 7.39 (3.36) hours sitting per weekday. Approximately half

For peer review only

For peer review only - http://bmjopen.bmj.com/site/about/guidelines.xhtml

of the participants (53.2%) spent 5-10 hours sitting, 30.3% spent 1-5 hours sitting, and 16.2% spent 10-16 hours sitting per weekday. Only 0.3 % of participants reported spending zero hours sitting per weekday. Sitting activity is classified as sedentary behaviour according to the Canadian Physical Activity and Sedentary Behaviour Guidelines [24].

For peer review only

Table 3. Participants Report of their Physical Activity Level during 7 days (n =1606)

Physical Activities	*None n (%)*	*1 day n (%)*	*2 days n (%)*	*3 days n (%)*	*4 days n (%)*	*5 days n (%)*	*6 days n (%)*	*7 days n (%)*	*p-values*
Vigorous physical activities [a]	987 (64)	120 (7.8)	123 (8)	114 (7.4)	49 (3.2)	54 (3.5)	23 (1.5)	71 (4.6)	<.001*
Moderate physical activities [b]	622 (39.9)	174 (11.2)	186 (11.9)	186 (11.9)	45 (2.9)	96 (6.2)	38 (2.4)	210 (13.5)	<.001*
Walk for at least 10 minutes at a time [c]	272 (17.4)	211 (13.5)	226 (14.4)	209 (13.3)	68 (4.3)	234 (14.9)	79 (5)	268 (17.1)	<.001*

[a] 65 participants did not answer this question, [b] 49 participants did not answer this question,
[c] 39 participants did not answer this question.
All p-values are computed by one sample Chisquare test, with d.f.=7.
* Significant at α=0.05 levels.

Most of the 1,606 participants (98.6%) believed both physical activity and eating a healthy diet were beneficial. However, less than half (45.4%) reported engaging in physical activity and only 34.5% reported eating healthy. Table 4 shows the participants' perceived level of physical activity and healthy eating.

Table 4. Level of Physical Activity and Healthy Eating (n=1604)

Perceived Level of Physical Activity and Healthy Eating	Yes n (%)	No n (%)	Sometimes n (%)	P-values
Do you engage in PA?	729 (45.4%)	466 (29.1%)	409 (25.5%)	<.001*
Do you think you eat healthy food?	553 (34.5%)	432 (26.9%)	618 (38.5%)	<.001*

All p-values are computed by one sample Chisquare test, with d.f.=2.
* Significant at α=0.05 levels.

Table 5 shows that for those categorized as Physical Activity Engaged had a higher percentage of normal weight compared with those who did not engage (31.8% versus 20.6%). Similarly, those who ate healthy had a higher percentage of normal weight compared with those who did not eat healthy (30.0% versus 21.2%). For those 'sometimes' PA engaged and 'sometimes' eating healthy, the percentage of normal weight are shown in between 'Yes' and 'No' groups. BMI category is statistically related to Physical Activity Engagement and Eating Healthy with both p <0.05.

Table 5: Association between BMI and Physical Activity Engagement and Eating Habits

BMI category	Physical Activity Engagement			P-value	Eating Healthy			P-value
	Yes n (%)	No n (%)	Sometimes n (%)		Yes n (%)	No n (%)	Sometimes n (%)	
underweight	18 (2.5)	10 (2.2)	16 (3.9)		17 (3.1)	8 (1.9)	19 (3.1)	
normal	230 (31.8)	95 (20.6)	107 (26.4)		164 (30.0)	91 (21.2)	178 (28.9)	
overweight	284 (39.3)	167 (36.1)	128 (31.5)		206 (37.7)	155 (36.1)	217 (35.3)	
obese	191 (26.4)	190 (41.1)	155 (38.2)	<.001*	160 (29.3)	175 (40.8)	201 (32.7)	0.003*

*significant at α=0.05 level.

For peer review only - http://bmjopen.bmj.com/site/about/guidelines.xhtml

Dietary Behavior

Nearly one third of participants ate fresh fruits (35.8%), green vegetables (31.8%), and other vegetables such as carrots, tomatoes, cucumber (44.1%) at least once daily (Table 6). Similarly, nearly one third of participants consumed protein products such as fish, beef, or chicken 2 to 4 times weekly. One fourth of the study participants drank full fat milk, 20.6% drank skimmed or low-fat milk, and nearly 50% consumed milk products more than once daily. More than one-quarter of the study participants ate pasta and cakes or pastries 2-4 times in a week, 40.6% had a meal with white bread daily, and 14.4% drank carbonated soda more than once daily.

For peer review only

Table 6. Food intake for daily meal (n =1606)

Food	Never n (%)	Seldom n (%)	1/week n (%)	2-4 /week n (%)	5-6 /week n (%)	1+ daily n (%)	P-values
Fruits & Vegetables							
Fresh fruits (apples, oranges, pears)[a]	20 (1.3)	123 (7.7)	165 (10.3)	469 (29.3)	251 (15.7)	572 (35.8)	<.001*
Green leafy vegetables[b] *(lettuce, cabbage, spinach)*	35 (2.2)	99 (6.2)	180 (11.2)	485 (30.3)	293 (18.3)	510 (31.8)	<.001
Other vegetables, carrots, tomatoes, cucumber)[c]	22 (1.4)	68 (4.2)	85 (5.3)	392 (24.4)	326 (20.3)	708 (44.1)	<.001*
Proteins							
Fish[d]	128 (8)	292 (18.3)	609 (38.1)	462 (28.9)	63 (3.9)	45 (2.8)	<.001*
Beef[e]	354 (22.3)	352 (22.1)	334 (21)	417 (26.2)	78 (4.9)	56 (3.5)	<.001*
Lamb[f]	197 (12.4)	279 (17.6)	397 (25.1)	532 (33.6)	91 (5.7)	88 (5.6)	<.001*
Chicken[a]	18 (1.1)	38 (2.4)	177 (11.1)	737 (46.1)	345 (21.6)	285 (17.8)	<.001*
Meat products (sausages, burgers, shawarma)[g]	276 (17.3)	496 (31.1)	341 (21.4)	305 (19.1)	110 (6.9)	67 (4.2)	<.001*
Eggs[g]	102 (6.4)	209 (13.1)	277 (17.4)	580 (36.4)	176 (11)	251 (15.7)	<.001*
Legumes (lentils, beans, peas)[h]	102 (6.4)	284 (17.7)	391 (24.3)	545 (33.9)	136 (8.5)	128 (8)	<.001*
Milk Products							
Milk, full fat[e]	430 (27)	234 (14.7)	115 (7.2)	245 (15.4)	174 (10.9)	393 (24.7)	<.001*
Milk, low fat or skimmed[i]	528 (33.1)	317 (19.9)	104 (6.5)	199 (12.4)	120 (12.5)	328 (20.6)	<.001*
Milk product (cheese, yogurt, milk drinks)[j]	38	90 (5.6)	100 (6.2)	393 (24.5)	266 (16.6)	716 (44.7)	<.001*

For peer review only

	(2.4)						
Carbohydrates							
Bread, white[c]	169 (10.6)	175 (10.9)	81 (5.1)	257 (16.1)	237 (14.8)	682 (42.6)	<.001*
Bread, whole meal/brown[k]	432 (27)	408 (25.5)	131 (8.2)	201 (12.6)	95 (5.9)	331 (20.7)	<.001*
Cereals (cornflakes, oatmeal)[l]	494 (30.9)	423 (26.5)	225 (14.1)	208 (13)	75 (4.7)	172 (10.7)	<.001*
Pasta (spaghetti, macaroni, noodles, grits)[j]	72 (4.5)	201 (12.5)	387 (24.1)	469 (29.3)	202 (12.6)	272 (17)	<.001*
Snack foods (potato chips, popcorn, chocolates…)[a]	211 (13.2)	385 (24.1)	307 (19.2)	368 (23)	135 (8.4)	194 (12.1)	<.001*
Cakes and pastries (cakes, biscuits, sweet pies)[a]	143 (8.9)	414 (25.8)	351 (21.9)	416 (26)	126 (7.9)	150 (9.4)	<.001*
Others							
Soft drinks (cola drinks)[c]	531 (33.2)	335 (20.9)	171 (10.7)	223 (13.9)	111 (6.9)	230 (14.4)	<.001*
Nuts (pistachio, cashew nuts)[m]	154 (9.7)	501 (31.5)	335 (21.1)	346 (21.8)	79 (5)	174 (11)	<.001*
Fruit Juices[d]	102 (6.4)	217 (13.6)	263 (16.4)	508 (31.8)	204 (12.8)	305 (19.1)	<.001*
Fruit drinks[a]	310 (19.4)	321 (20.1)	131 (8.2)	373 (23.3)	158 (9.8)	307 (19.1)	<.001*
Coffee/tea[c]	77 (4.8)	82 (5.1)	51 (3.2)	142 (8.9)	216 (13.5)	1033 (64.5)	<.001*

[a] 6 participants did not answer this question, [b] 4 participants did not answer this question, [c] 5 participants did not answer this question, [d] 7 participants did not answer this question, [e] 15 participants did not answer this question, [f] 22 participants did not answer this question, [g] 11 participants did not answer this question, [h] 20 participants did not answer this question, [I] 10 participants did not answer this question, [j] 3 participants did not answer this question, [k] 8 participants did not answer this question, [l] 9 participants did not answer this question, [m] 17 participants did not answer this question.

All p-values are computed by one sample Chisquare test, with d.f.=5.

* Significant at α=0.05 levels.

For peer review only - http://bmjopen.bmj.com/site/about/guidelines.xhtml

Due to ordinal nature of variables in Tables 3, 4 and 6, Spearman rank correlation coefficients were computed to determine their linear relationships. The results reveal that there is little or no relationship (|r| < .25) among the variables between Tables 3 and 4 (except with Physical Activities Engagement), tables 3 and 6, and tables 4 and 6. However, there is a fair degree of relationship between intensity of physical exercise (variables in Table 3) with Physical Activities Engagement (variable in Table 4) with coefficients ranging from 0.298 to 0.485 (Table 7).

Table 7. Spearman Rank Correlation Coefficients between Physical Activities Engagement and Intensity of Physical Exercise

	Vigorous physical exercise	Moderate physical exercise	Walk for at least 10 minutes at a time
Physical Activities Engagement	0.485 (p<.001*,n=1539)	0.298 (p<.001*, n=1555)	0.348 (p<.001*,n=1565)

*Significant at α=0.05 levels.

For peer review only

Despite participants' perceived low level of being physically active (45.4%) and eating healthy (34.5%), the majority of participants found that engaging in physical activity (88.7%) and eating healthy foods (74.3%) were pleasant. Furthermore, 98.6% of participants believed that being active and eating healthy are beneficial for health. More than half strongly agreed that engaging in physical activity (73.1%) and eating health food (66.6%) would help maintain good health. Out of 1606, 75.9% of participants communicated that they would engage in physical activity and 75.5% said they would eat healthy foods within the next month. Many were willing to continue to be physically active for the next 6 months (81.5%) and eat healthy foods (79.2%).

Reported Health Status

Nearly half of the study participants (49.1%) reported good health, while the remaining cited excellent (26.2%), fair (19.3%), or poor (4.5%) health. Most participants did not have a medical history of heart attack, cancer, psychiatric illness, polycystic ovarian syndrome, or fractures. Overall, 10% to 13% of the participants reported a high blood cholesterol level, high blood pressure, diabetes, obesity, or bowel problems (Table 8).

Table 8. Medical History of Study Participants – (n =1606)

MEDICAL HISTORY	YES N (%)	NO N (%)	P-VALUES
HIGH BLOOD PRESSURE [A]	206 (12.9)	1389 (87.1)	<.001*
BOWEL PROBLEMS [C]	195 (12.2)	1409 (87.8)	<.001*
HIGH BLOOD	183 (11.5)	1411 (88.5)	<.001*

For peer review only

CHOLESTEROL [B]			
DIABETES [D]	180 (11.2)	1422 (88.8)	<.001*
OBESITY [E]	162 (10.1)	1443 (89.9)	<.001*
ALLERGIES [F]	146 (9.1)	1457 (90.9)	<.001*
STOMACH PROBLEMS [E]	130 (8.1)	1475 (91.9)	<.001*
ASTHMA [E]	73 (4.5)	1532 (95.5)	<.001*
FRACTURES [E]	46 (2.9)	1559 (97.1)	<.001*
POLYCYSTIC OVARIAN SYNDROME [D]	45 (2.8)	1557 (97.2)	<.001*
OSTEOPOROSIS [G]	44 (2.8)	1553 (97.2)	<.001*
PSYCHIATRIC ILLNESS [E]	12 (0.7)	1593 (99.3)	<.001*
HEART ATTACK [C]	10 (0.6)	1594 (99.4)	<.001*
CANCER	2 (0.1)	1604 (99.9)	<.001*
STROKE [C]	1 (0.1)	1603 (99.9)	<.001*

[a] 11 participants did not answer this question, [b] 12 participants did not answer this question, [c] 2 participants did not answer this question, [d] 4 participants did not answer this question, [e] 1 participants did not answer this question, [f] 3 participants did not answer this question, [g] 9 participants did not answer this question,
All p-values are computed by one sample Chisquare test, with d.f.=1.
* Significant at α=0.05 levels

For PA engagement prediction, a list of potential predictors: living area, marital status, nationality, age group, education, health status, household income and sex were chosen. Forward stepwise (Wald χ2, pin=0.05, pout=0.10) logistic regression method was used. Only marital status, age group, sex and education are statistically significant in predicting PA Engagement.

For peer review only

Table 9 summarizes multivariate forward stepwise logistic regression analyses results with significant independent factors that may be used as indicators to predict participant engagement in physical activities. Married participants had 0.676 times (p=0.021), older people had 0.679 times (p=0.009), and females had 0.348 times (p<0.001) the odds of physical activities engagements. Level of education was also a statistical significant predictor of physical activities engagements ($\chi2$ (5) =11.124, p=0.049).

Table 9. Association between Significant Factors (results from forward stepwise logistic regression) and Physical Activities Engagement

	Crude OR (95% CI)	P value	Adjusted OR (95% CI)	P value
Predictors of Physical Activities Engagement				
Marital Status				
Not married (reference)			1.0	
Married	0.54(0.40-0.71)	<.001*	0.68 (0.49 – 0.94)	0.021*
Age group				
18-39 (reference)	1.0		1.0	
40 & older	0.57(.44-.74)	<.001*	0.68 (0.51 – 0.91)	0.009*

For peer review only

	Crude OR (95% CI)	P value	Adjusted OR (95% CI)	P value
Sex				
Male (reference)	1.0		1.0	
Female	0.38(0.29-0.49)	<.001*	0.35 (0.27 – 0.46)	<0.001*
Education of Participant (Wald $\chi^2(5)$=11.12)				0.049*
≤Primary/intermediate school(reference)	1.0		1.0	
Primary/Junior High	.72(0.20-0.26)	0.607	0.71 (.19 – 2.65)	0.615
High School	1.51(0.45-5.12)	0.508	1.04(.29 - 3.72)	0.949
Trade	0.39(0.90-1.65)	0.090	0.25(.06 -1.13)	0.072
University	1.03(0.31-3.47)	0.961	0.82(.23 - 2.89)	0.761
Other	1.19(0.34-4.20)	0.792	0.98(.27 – 3.62)	0.978

Model Summary		
-2 Log likelihood	Cox & Snell R Square	Nagelkerke R Square
1333.527	0.080	0.113

For peer review only

Similarly, for Eating Healthy Food, we used the same list of potential predictors as in predicting PA Engagement. Variables such as living area, marital status, nationality, age group, education, health status, household income and sex were selected. Forward stepwise (Wald $\chi2$, pin=0.05, pout=0.10) logistic regression method was used. Only age group, health status and living area were statistically significant in predicting Eating Healthy Food.

Table 10 summarizes multivariate forward stepwise (Wald $\chi2$, pin=0.05, pout=0.10) logistic regression analyses results with significant independent factors that may be used as indicators to predict participants eating healthy food. Older people had 0.68 times (p=0.005) and people who lived in Al-Wakra (South of Qatar) had 0.57 times (p=0.007) the odds of eating healthy food. Living area is a statistically significant predictor of eating healthy food ($\chi2$ (2) =8.35, p=0.007). Health status is also a statistically significant predictor with one unit improvement in health status having 1.35 times (p=.002) the odds of eating healthy food.

For peer review only

Table 10. Association between Significant Factors (results from forward stepwise logistic regression) and 'do you think you eat healthy food?'

Predictors of 'do you think you eat healthy food?'	Crude OR(95%)	P value	Adjusted OR (95% CI)	P value
Age group				
18-39 (reference)	1.0		1.0	
40 & older	0.68(0.52-0.89)	0.004*	0.68 (0.52 – 0.89)	0.005*
Living Area (Wald $\chi^2(2)$=8.09)				0.018*
Al-Doha (reference)	1.0		1.0	
Al-Wakra (South of Qatar)	0.57(0.38-0.86)	0.008*	0.57 (0.38 – 0.85)	0.007*
Al-Khor/ Al Thakhira (North of Qatar)	1.10(0.74-1.63)	0.632	1.13 (0.76 – 1.68)	0.558
Health Status	1.35(1.12-1.63)	0.002*	1.35(1.11-1.63)	0.002*

Model Summary		
-2 Log likelihood	Cox & Snell R Square	Nagelkerke R Square
1315.497	0.021	0.031

For IPAQ groups prediction, due to ordinal natural of IPAQ groups, ordinal logistic regression was supposed to be used to determine the relationship between IPAQ groups and the following variables: Living area, marital status, nationality, age group, education, health status, household income and sex. However, it did not satisfy the assumption of parallel lines for ordinal logistic regression (-2 log likelihood =1728.24, $\chi2(22)$=52.90, p<.001). Therefore, we combined Moderate and High IPAQ groups into one group. Forward stepwise (Wald $\chi2$, pin=0.05, pout=0.10) logistic regression was performed on two groups (group 1: low, group 2: moderate & high combined).

Table 11 summarizes forward stepwise logistic regression results in predicting 'Moderate & High combined'. Out of this set of potential predictors, only Marital Status and

For peer review only

Health status were statistically significant in predicting moderate & high IPAQ group. Married participants had 0.75 times (p=.042) the odds of 'moderate & high' and one unit improvement in Health Status which had 1.41 times (p=.001) the odds of having 'moderate & high' physical activity.

Table 11. Association between Significant Factors (results from forward stepwise logistic regression on IPAQ group (Low versus 'Moderate & High')

Predictors of IPAQ (Low versus 'moderate & high')	Crude OR (95%)	P value	Adjusted OR (95% CI)	P value
Marital Status				
Not married (reference)	1.0		1.0	
Married	0.75(0.54-1.02)	0.069	0.72 (0.52 – 0.99)	0.042*
Health Status	1.41(1.15-1.74)	0.001*	1.43(1.16-1.77)	0.001*0.001*

Model Summary		
-2 Log likelihood	Cox & Snell R Square	Nagelkerke R Square
1145.37	0.013	0.020

Discussion

In this cross-sectional community-based survey study, we examined the level of physical activity, dietary habits, and health status among adult Arabic speakers living in the State of Qatar. Our findings highlight the low physical activity levels in study participants. It is known that low physical activity level in the Middle East is related to its weather condition [25, 26] i.e., the environmental factor of a hot desert climate is a barrier for people to engage in exercise and participate in outdoor activities. In summer, the temperature is very high in Qatar (30–50 degree Celsius), which restricts outdoor activities such as walking, cycling, and jogging. Other reported barriers include lack of interest, motivation and information about the

For peer review only

benefits of exercise, stress, excessive internet and computer usage, and lack of accessible exercise facilities [27, 28].

Congruent with previous research and surveys done in Qatar and GCC countries, that were discussed in the beginning of this report, the findings of this study suggests that the Qatar adult population's physical activity is insufficient to meet the amount recommended by the WHO and Qatar Physical Activity Guidelines. As well, it was noted that there is a difference in the level of vigorous (i.e., running) and mild (i.e., casual walking) activity between male and female participants. Previous studies have reported similar findings with females being less involved in regular physical activity [29, 30]. Similarly, recent work in Qatar revealed that males were more physically active than females, both for walking (74.6% of males versus 55.3% of females) and intense sports (16.5% of males versus 8.9% of females) [31]. An Omani study found that females were significantly more inactive compared to male participants (76.9% versus 33.3%) [32]. Direct comparisons across studies are complicated due to multiple factors such as methodological variations, ethnicity, geographical location and cultural values [33]. However, low the level of involvement of women in regular physical activities in Qatar is possibly related to cultural and social norms (i.e., women in many Islamic countries need to be accompanied by a male family member when going outdoors), caregiving responsibilities, the need to wear an Abaya in public, and a general lack of social support for exercise [8, 25, 28, 34-37]. Even though the present study's data indicated that female participants are less likely to be physically active, older and married women had slightly higher odds of being involved in physical activity. We anticipated that this might be related to the fact that older married women are more physically involved in family domestic activities such as house chores and taking care of children and other family members.

For peer review only - http://bmjopen.bmj.com/site/about/guidelines.xhtml

Sitting has been highlighted as a specific marker of sedentary behaviors [38]. In this study, it was alarming that slightly more than half (53.2%) of the participants spent 5-10 hours sitting per day. In contrast, a recently published research article reported that the overall median time spent in sedentary activities was 179 minutes (2.98 hours) per day among Qatari nationals [26]. This discrepancy in findings is perhaps due to diverse participant characteristics. Our study has more participants with different ethnocultural backgrounds than just Qatari nationals. More sitting time and insufficient physical activity are significantly associated with higher health risk factors such as abdominal obesity, dyslipidemia, hyperglycemia and hypertension among the adult population [39].

In the present study, about two thirds of participants were either overweight or obese with a mean BMI of 28.03 kg/m^2. Our findings are consistent with a previous study conducted in Qatar, where 70.1% of participants had a BMI equal to or above 25 kg/m^2 (classified as overweight and obese) [31]. The prevalence of obesity in GCC countries is among the highest in the world. According to the Global status report in 2014 [10], rates of obesity reached more than 37% in the UAE, almost 40% in Kuwait, and more than 42% in Qatar. Similarly, high Body Mass Index in Saudi Arabia (34.7%) and in Lebanon (31.9%) have been reported [40].

The rapid increase in wealth and subsequent development of Arab countries has led to changes in lifestyle. One of the many consequences of region development secondary to abundant oil resources, has been a significant change in eating habits. The GCC's former Mediterranean-style diet of traditional products (e.g., dates, vegetables, wheat) have been replaced by a reliance on fast foods that are dominated by refined and processed meals heavy in sugar and other carbohydrates [41]. This might help to explain our findings that older participants who lived outside of the capital had slightly higher odds of eating healthy because they might be less likely to adopt or access Western-style diet. There is strong

For peer review only - http://bmjopen.bmj.com/site/about/guidelines.xhtml

evidence for the benefits of healthy diet in primary prevention of major cardiovascular events, and reducing the risk of diabetes among people with high cardiovascular risk [42, 43]. In a recent qualitative study, Donnelly and colleagues [28] examined socio-cultural factors that influenced the healthy lifestyles (i.e., diet, physical activity, non-smoking) of 50 Arabic women with heart diseases living in Qatar. The participants reported that their diets tended to be high in salt, sugar, and fats. Their diets were also influenced by traditional cultural beliefs and values. For example, women were often invited to each other homes where sweets and coffee were served. The women ate foods that were offered because refusing to eat the food would be considered socially unacceptable behavior. Furthermore, Donnelly and colleagues [34] reported that social support, cultural values, religion, hot desert climate, heart disease, changing socio-demographic and economic conditions impacted both positively and negatively on the ability of these women to pursue a healthy lifestyle.

Overall, participants' dietary habits in this study were found to be unhealthy: 40.6% ate white bread daily and had pasta and cakes or pastries 2-4 times per week. Non-carbohydrate-containing foods such as fruits, legumes, vegetables, and minimally processed whole grains are healthy and cardio-metabolic protective, while foods rich in refined grains, e.g., white bread, white rice, crackers, cereals, bakery desserts, starches, and added sugars are associated with weight gain [20]. A study conducted in Oman reported few gender differences with respect to eating habits, except in dairy and meat consumption where 62.5% and 55.5% of men consumed more than 3 servings, compared to 18.78 % and 35.2% of women, respectively [32]. Dietary behaviors are mainly influenced by individual factors (taste preferences, dietary knowledge, stress, body image, former eating habits and physical activity level) and environmental factors (availability and accessibility, costs of food products) [44]. Given that more females than males were in the categories of underweight and obese class III in our study, facilitating Arab female engagement in healthier lifestyle

For peer review only - http://bmjopen.bmj.com/site/about/guidelines.xhtml

needs to be emphasized. There is a need to develop collaboration among health care professionals, academics, public health professionals, and policy makers to improve the dietary behaviors and lifestyle of the population. In the UK, the National Institute for Health and Care Excellence (NICE) recommends that primary care practitioners deliver brief physical activity advice to lethargic adults and follow up on outcomes at subsequent appointments [45].

Conclusion

We conclude that there are insufficient levels of physical activity and poor dietary behavior among the population in Qatar. Insufficient levels of physical activity and unhealthy dietary present a great health risk. There is a need to develop a nationwide health promotion program that aims at increased knowledge about the benefits of being physically active and eating healthy. Awareness of and adherence to Qatar physical activity guidelines and the new Arabic dietary guidelines should be encouraged by health care policy makers and health care providers. The findings of this study might provide insights and information necessary for the development of public health policies and promotion programs in Qatar and in the Middle East region.

Study Limitations

The present study nonetheless has multiple limitations that should be considered when interpreting the results. Non-probability convenience sampling limits the ability to generalise the findings from this study. As well, the findings of this study might not be valid for generalization to other population segments of different race/ethnicity and socio-economic status due to geographic/cultural differences. However, the findings are relevant to a population with similar ethnic and cultural backgrounds. In this study, participants were 18 years of age and over. Younger participants (age <18 years) might possibly give different

For peer review only

perspectives in regards to their physical activity and dietary behaviours. In addition, information on dietary habits in the present study was based on the frequency of consumption of food items without much consideration of quantity or portion size. Future research is needed to investigate the association between Arabs' attitude, perceived behavioural control, and their physical activity level and dietary habit.

Acknowledgments

This study and publications was made possible by a grant from Qatar National Research Fund under its National Priority Research Program (**NPRP 6 – 049 – 3 – 009)** for which we are very grateful. Its contents are solely the responsibility of the authors and do not necessarily represent the official views of Qatar National Research Fund.

We thank our study participants who accepted to join us and share their experiences. We hope through this report we repay them for their insight and trust. We give special thanks to the staff at universities and college, and Primary Health Care Centers who helped us to recruit the research participants, to our project coordinator, Shima Sharara and to our research assistants: Nagla HagOmer, Sara Saeed, Samira Mouhamed. We also thank Muhammad Siddiqui for his assistance with this report.

Authors' contributions

TTD: Contributed to the conception and design of the study and the acquisition, analysis, and interpretation of data, drafted the manuscript, and gave final approval of the manuscript version submitted for publication.

TF: Contributed to the conception and design of the study and the acquisition of data, revised the manuscript, and gave final approval of the manuscript version submitted for publication.

AA: Contributed to the conception and design of the study and the acquisition of data, reviewed the manuscript critically for content, and gave final approval of the manuscript version submitted for publication.

Funding

This study was made possible by a grant from the Qatar National Research Fund under its National Priorities Research Program (**NPRP 6 – 049 – 3 – 009**). Its contents are solely the responsibility of the authors and do not necessarily represent the official views of the Qatar National Research Fund.

Conflict of interest

The authors report no relationships that could be construed as a conflict of interest.

For peer review only

Ethics approval

The ethical approvals were obtained from several ethics review boards (IRBs) which include: Hamad Medical Corporation/Weill Cornell Institutional Review Board (IRB NO: 13-00051), the University of Calgary's Conjoint Health Research Ethics Board (REB13-0347), Qatar Primary Health Care Research Committee (Reference NO: PHCC/RC/14/02/004), Qatar University (QU-IRB 244-E/13), College of North Atlantic in Qatar (CNAQ Approval NO: 2015-3), and Qatar Supreme Council of Health (SCH-A-UCQ-050).

Data sharing statement

No additional data are available.

References

1. Ding D, Lawson K, Kolbe-Alexander T, Finkelstein E, Katzmarzyk P, van Mechelen W, et al. The economic burden of physical inactivity: a global analysis of major non-communicable diseases. The Lancet. 2016;388(10051):1311-24. doi: https://doi.org/10.1016/S0140-6736(16)30383-X.

2. Lee IM, Shiroma EJ, Lobelo F, Puska P, Blair SN, Katzmarzyk PT. Effect of physical inactivity on major non-communicable diseases worldwide: an analysis of burden of disease and life expectancy. The Lancet. 2012;380(9838):219-29. doi: https://doi.org/10.1016/S0140-6736(12)61031-9.

3. Booth FW, Roberts CK, Laye MJ. Lack of exercise is a major cause of chronic diseases. . Compr Physiol. 2012;2(2):1143–211. doi: doi: 10.1002/cphy.c110025.

4. Yusuf S, Hawken S, Ôunpuu S, Dans T, Avezum A, Lanas F, et al. Effect of potentially modifiable risk factors associated with myocardial infarction in 52 countries (the INTERHEART study): case-control study. The Lancet. 2004;364(9438):937-52. doi: doi.org/10.1016/S0140-6736(04)17018-9.

5. World Health Organization (WHO) World Health Statistics—2014. World Health Organization. Available from: http://apps.who.int/iris/bitstream/10665/112738/1/9789240692671_eng.pdf?ua=1.

6. Global recommendations on physical activity for health The World Health Organization website. . [cited 2012 October]. Available from: http://www.who.int/dietphysicalactivity/factsheet_recommendations/en/2011.

For peer review only

7. Al-Bibi KW. The State of Qatar national physical activity guidelines. Qatar University. Available from: https://www.namat.qa/NamatImages/Publications/75/QATAR%20PA%20GUIDLINE%20ENGLISH.PDF.

8. Mabry R, Reeves M, Eakin E, Owen N. Evidence of physical activity participation among men and women in the countries of the Gulf cooperation council: a review. Obes Rev. 2010;11(06):457-64. doi: 10.1111/j.1467-789X.2009.00655.x.

9. World Health Organization. Qatar STEPwise approach to chronic disease risk factor surveillance. Available from: http://www.who.int/ncds/surveillance/steps/qatar/en/.

10. Global status report on noncommunicable diseases. Geneva: World Health Organization. 2014. Available from: http://apps.who.int/iris/bitstream/10665/148114/1/9789241564854_eng.pdf?ua=1.

11. Ramirez FD, Chen Y, Di Santo P, Simard T, Motazedian P, Hibbert B. Association between self□reported potentially modifiable cardiac risk factors and perceived need to improve physical health: a population□based study. J Am Heart Assoc. 2017;6.

12. Collese T, Nascimento-Ferreira M, de Moraes A, Rendo-Urteaga T, Bel-Serrat S, Moreno L, et al. Role of fruits and vegetables in adolescent cardiovascular health: a systematic review. Nutr Rev. 2017;ahead of print. doi: 10.1093/nutrit/nux002.

13. Food and Agriculture Organization of the United Nations. Food-based dietary guidelines - Qatar. Available from: http://www.fao.org/nutrition/education/food-dietary-guidelines/regions/countries/qatar/en/.

14. Badran M, Laher I. Obesity in arabic-speaking countries. J Obes. 2011;2011:686430. doi: 10.1155/2011/686430. PubMed PMID: 22175002; PubMed Central PMCID: PMCPMC3228340.

15. Mehio Sibai A, Nasreddine L, Mokdad AH, Adra N, Tabet M, Hwalla N. Nutrition transition and cardiovascular disease risk factors in Middle East and North Africa countries: reviewing the evidence. Ann Nutr Metab. 2010;57(3-4):193-203. Epub 2010/11/23. doi: 10.1159/000321527. PubMed PMID: 21088386.

For peer review only

16. Ng SW, Zaghloul S, Ali H, Harrison G, Yeatts K, El Sadig M, et al. Nutrition transition in the United Arab Emirates. Eur J Clin Nutr. 2011;65(12):1328-37. Epub 2011/07/21. doi: 10.1038/ejcn.2011.135. PubMed PMID: 21772317; PubMed Central PMCID: PMCPMC3304306.

17. Qatar Statistics Authority, Ministry of development planning and statistics, population and social statistics. 2016.

18. Institute of Chartered Accountants in England and Wales (ICAEW) Economic insight: Middle East. Quarterly briefing Q3. 2016. Available from: http://www.icaew.com/-/media/corporate/files/technical/economy/economic-insight/middle-east/me-q3-2016-web.ashx

19. World Health Organisation: Obesity and overweight 2016. Available from: http://www.who.int/mediacentre/factsheets/fs311/en/.

20. Mozaffarian D, Hao T, Rimm E, Willett W, Hu F. Changes in diet and lifestyle and longterm weight gain in women and men. N Engl J Med. 2011;364:2392-404.

21. WG C. Sampling Technique. 3rd ed ed: John Wiley & Sons: New York; 1997.

22. Craig C, Marshall A, Sjöström M, Bauman A, Booth M, Ainsworth B, et al. International physical activity questionnaire: 12-country reliability and validity. Med Sci Sports Excer. 2003;35(8):1381-95.

23. IPAQ scoring protocol. . Available from: https://sites.google.com/site/theipaq/scoring-protocol.

24. Canadian Society for Exercise Physiology. Canadian physical activity guidelines Canadian sedentary behaviour guidelines. Available from: https://www.albertaquits.ca/files/AB/files/library/CSEP_Guidelines_Handbook.pdf.

25. Ali HI BL, Bernsen RM. Barriers and facilitators of weight management: perspectives of Arab women at risk for type 2 diabetes. Health Soc Care Community. 2010;18(2):219-28. doi: 10.1111/j.1365-2524.2009.00896.x.

For peer review only

26. Kahan D. Arab American college students' physical activity and body composition: reconciling Middle East-West differences using the socioecological model. Res Q Exerc Sport 2011;82(1):118-28.

27. Benjamin K, Donnelly T. Barriers and facilitators influencing the physical activity of Arabic adults: A literature review. Avicenna. 2013;2013(1):8. doi: 10.5339/avi.2013.8.

28. Donnelly TT, Al Suwaidi J, Al Bulushi A, Al Enazi N, Yassin K, Rehman AM, et al. The influence of cultural and social factors on healthy lifestyle of Arabic women. Avicenna. 2011;(2011):3. doi: 10.5339/avi.2011.3.

29. Al-Hazzaa HM, Al-Sobayel HI, Abahussain NA, Qahwaji DM, Alahmadi MA, Musaiger AO. Association of dietary habits with levels of physical activity and screen time among adolescents living in Saudi Arabia. J Hum Nutr Diet. 2014;27 Suppl 2:204-13. doi: 10.1111/jhn.12147. PubMed PMID: 23889093.

30. Fernandes RA, Christofaro DF, Casonatto JF, Kawaguti SS, Ronque ERV, Cardoso JR, et al. Cross-sectional association between healthy and unhealthy food habits and leisure physical activity in adolescents. J Pediatr (Rio J). 2011;87(3):252-6. doi: doi:10.2223/JPED.2093.

31. Al-Thani MH A-TA, Al-Chetachi WF, Khalifa SAH, Akram H, Poovelil BV, Almalki BA, Bakri AH, Arora P, Badawi A. Dietary and nutritional factors influencing obesity in Qatari adults and the modifying effect of physical activity. J Obes Weight-Loss Medic. 2015;1(1):1-7.

32. Kilani H, Al-Hazzaa H, Waly M, Musaiger A. Lifestyle Habits: Diet, physical activity and sleep duration among Omani adolescents. Sultan Qaboos Univ Med J. 2013;13(4):510–9.

33. Hamer M, Brunner E, Bell J, Batty G, Shipley M, Akbaraly T, et al. Physical activity patterns over 10 years in relation to body mass index and waist circumference: the Whitehall II cohort study. Obesity. 2013;21(12):E755-61.

34. Donnelly TT, Al Suwaidi J, Al Bulushi A, Al Enazi N, Yassin K, Rehman AM, et al. Qatari women living with cardiovascular diseases-challenges and opportunities to engage in

For peer review only

healthy lifestyles. Health Care Women Int. 2012;33(12):1114-34. doi: 10.1080/07399332.2012.712172. PubMed PMID: 23153347.

35. Amin TT, Suleman W, Ali A, Gamal A, A. AW. Pattern, prevalence, and perceived personal barriers toward physical activity among adult Saudis in Al-Hassa, KSA. J Phys Act Health. 2011;8(6):775-84.

36. Qahoush R, Stotts N, Alawneh M, Froelicher E. Physical activity in Arab women in Southern California. Eur J Cardiovasc Nurs. 2010;9(4):263-71. doi: 10.1016/j.ejcnurse.2010.03.002.

37. Shuval K, Weissblueth E, Araida A, Brezis M, Faridi Z, Ali A, et al. The role of culture, environment, and religion in the promotion of physical activity among Arab Israelis. Prev Chronic Dis. 2008;5(3):A88. doi: D - NLM: PMC2483551 EDAT- 2008/06/19 09:00 MHDA- 2008/08/08 09:00 CRDT- 2008/06/19 09:00 AID - A88 [pii] PST - ppublish.

38. Owen N, Bauman A, Brown W. Too much sitting: a novel and important predictor of chronic disease risk? Br J Sports Med. 2009;43(2):81–3.

39. Chu AHY, Moy FM. Joint association of sitting time and physical activity with metabolic risk factors among aiddle-aged Malays in a developing country: a cross-sectional study. Bayer A, ed. . PLoS ONE 2013;8(4):e61723. doi:10.1371/journal.pone.0061723.

40. Confronting obesity in the Middle East cultural, social and policy challenges. The economist intelligence unit limited 2016. Available from: https://www.eiuperspectives.economist.com/sites/default/files/ConfrontingobesityintheMiddleEast_0.pdf

41. Gerber M, Hoffman R. The Mediterranean diet: health, science and society. Br J Nutr. 2015;113(2):S4-10.

42. Estruch R, Ros E, Salas-Salvad J, Covas M, Corella D, Arós F, et al. Primary prevention of cardiovascular disease with a Mediterranean diet. N Engl J Med. 2013;368(14):1279-90. doi: 10.1056/NEJMoa1200303.

For peer review only

43. Salas-Salvadó J, Bulló M, Estruch R, Ros E, Covas M, Ibarrola-Jurado N, et al. Prevention of diabetes with Mediterranean diets: a subgroup analysis of a randomized trial. Ann Intern Med. 2014;160(1):1-10. doi: 10.7326/M13-1725.

44. Deliens T, Clarys P, De BI, Deforche B. Determinants of eating behaviour in university students: a qualitative study using focus group discussions. BMC Public Health. 2014;14(1):53.

45. National Institute for Health and Care Excellence. Physical activity: brief advice for adults in primary care. NICE Public Health Guidance 44. National Institute for Health and Care Excellence. 2013. Available from: https://www.nice.org.uk/guidance/ph44.

Figure 1. Gulf coast of the Arabian Peninsula

Figure 2: Distribution of BMI among participants

For peer review only

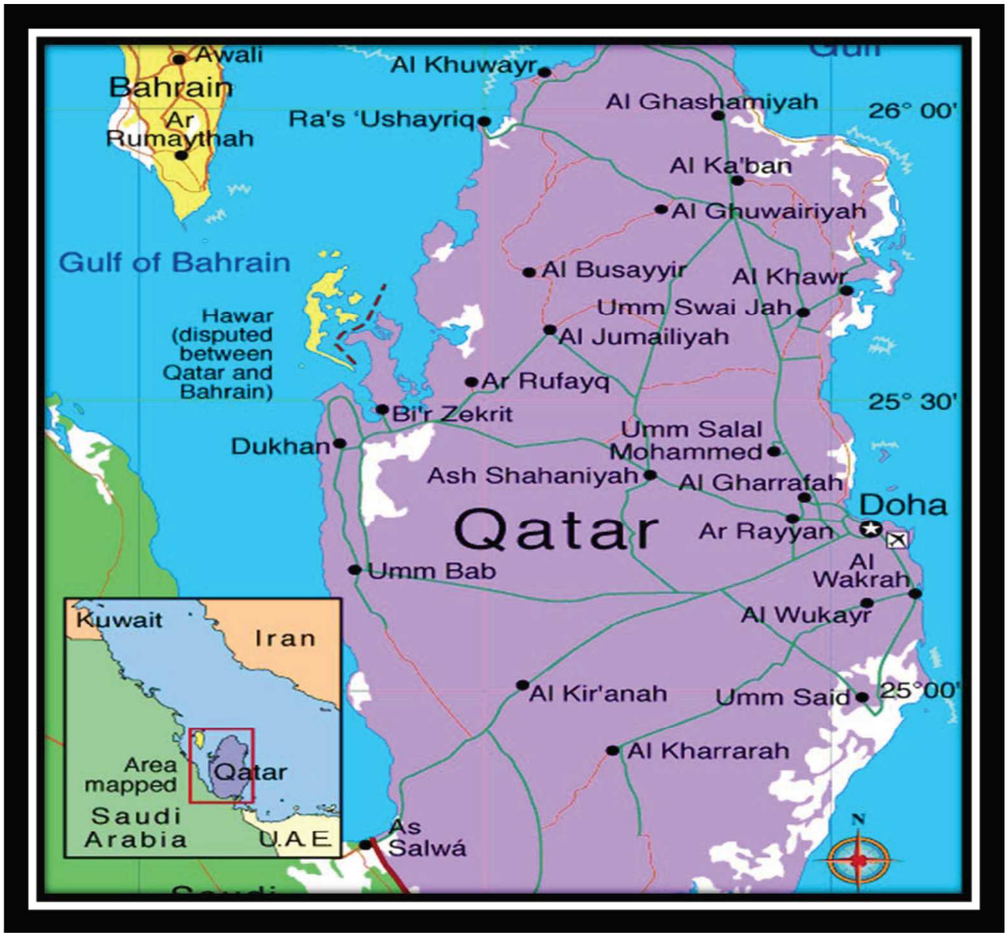

Figure 1. Gulf coast of the Arabian Peninsula

147x136mm (300 x 300 DPI)

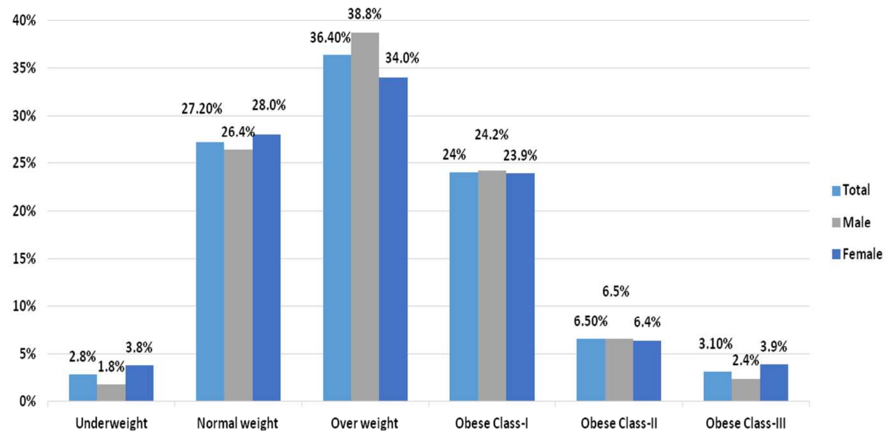

Figure 2: Distribution of BMI among participants

152x83mm (300 x 300 DPI)
